# Antimicrobial Peptides, Bacteriocins and Mycocins as Natural Antimicrobials: Applications in Food Safety, Agriculture and Healthcare

**DOI:** 10.3390/antibiotics15070649

**Published:** 2026-06-30

**Authors:** Patrícia Branco, Elisabete Muchagato Maurício, Luís R. Raposo, Catarina Roma-Rodrigues

**Affiliations:** 1 BIORG—Bioengineering and Sustainability Research Group, Lusófona University, Av. Campo Grande 376, 1749-024 Lisbon, Portugal; luismrraposo@gmail.com (L.R.R.); catarina.rodrigues@ulusofona.pt (C.R.-R.); 2 Linking Landscape, Environment, Agriculture and Food (LEAF), Associated Laboratory TERRA, Instituto Superior de Agronomia, University of Lisbon, Tapada da Ajuda, 1349-017 Lisbon, Portugal

**Keywords:** bacteriocins, antimicrobial peptides, mycocins, biopreservation, food safety, peptide-based compounds, microbial natural products, biopesticides, biofungicides

## Abstract

The growing concern over antimicrobial resistance and the increasing demand for safer and more sustainable antimicrobial strategies have driven extensive research into peptide-based natural antimicrobials. This review focuses specifically on antimicrobial peptides (AMPs), bacteriocins and mycocins as peptide- or proteinaceous antimicrobial compounds with potential applications as active ingredients, biopreservatives and antimicrobial tools. These compounds exhibit activity against spoilage and pathogenic microorganisms and are increasingly being explored in food safety, agriculture, cosmetics, animal health and human healthcare. AMPs, bacteriocins and mycocins act through diverse and sometimes overlapping mechanisms, including membrane disruption, pore formation, inhibition of cell wall biosynthesis, interference with intracellular targets, induction of oxidative stress and modulation of host or microbial responses. These mechanisms support their potential use in food biopreservation, crop protection, biofungicide and biopesticide development, topical antimicrobial formulations, cosmetic preservation, antibiofilm strategies and adjunctive therapeutic approaches. Recent advances in encapsulation, peptide engineering, recombinant production, nanodelivery and combination strategies with conventional antibiotics, hurdle technologies or other natural antimicrobials have improved the stability, bioavailability and antimicrobial efficacy of these compounds in experimental systems. However, broader translation remains limited by several major challenges. These include proteolytic degradation, reduced stability in complex matrices, context-dependent antimicrobial activity, possible toxicity, resistance development, high production and purification costs, formulation difficulties, scale-up limitations and regulatory constraints. Further validation is also needed regarding safety, microbiome impact, environmental fate and performance under realistic food-preservation, agricultural, cosmetic and clinical conditions. This review summarises and compares the diversity, mechanisms, applications and translational challenges of AMPs, bacteriocins and mycocins across food safety, sustainable agriculture, cosmetics, animal health and healthcare. It also discusses the main challenges that must be addressed before broader translation, including resistance risk, stability, formulation, scale-up, safety assessment and regulatory approval.

## 1. Introduction

Since their discovery, antibiotics have played a central role in the treatment of bacterial infections in humans and animals and have also been used in agricultural and food-production systems. However, their extensive and sometimes inappropriate use across these interconnected sectors has contributed to the emergence and dissemination of antimicrobial resistance, which now represents a major global public health threat [1,2,3,4]. It is estimated that if this trend remains uncontrolled, antimicrobial resistance may contribute to millions of deaths annually by 2050 [5]. In parallel, antibiotic residues and unmetabolized compounds released into the environment contribute to antibiotic pollution and may affect non-target organisms, including aquatic organisms, plants and soil-associated communities [2,6,7]. It is therefore essential to develop safer, more targeted and more sustainable antimicrobial strategies. A wide range of natural antimicrobial compounds has been investigated as potential alternatives to conventional antibiotics, including polyphenols, terpenoids, alkaloids, organosulfur compounds and bioactive products derived from plants and bee products, such as propolis, honey, garlic and onion extracts. These natural compounds exhibit antimicrobial, antioxidant and preservative properties and have shown potential applications in the food, agricultural, cosmetic and pharmaceutical sectors [8,9,10]. However, among the diverse natural antimicrobial strategies currently under investigation, biologically produced antimicrobial peptides and proteins have emerged as particularly promising candidates due to their potency, specificity and environmental compatibility. In this context, AMPs, bacteriocins and mycocins are gaining increasing attention because of their natural origin, structural diversity, biodegradability and antimicrobial activity [11].

These compounds include AMPs produced by diverse organisms, with bacteriocins referring specifically to antimicrobial peptides produced by bacteria and mycocins referring mainly to antimicrobial peptides or proteinaceous toxins produced by yeasts and other fungi. Together, they contribute naturally to microbial competition and have been exploited as food biopreservatives, antimicrobial ingredients and potential alternatives or adjuncts to conventional antibiotics [12,13]. Their safety profile, stability under specific processing conditions and activity against foodborne, clinical and environmental pathogens support their potential application in food, agriculture, cosmetics and healthcare [14,15]. However, this potential should be considered alongside important translational limitations, including possible cytotoxicity, haemolytic activity, reduced stability in complex environments, emergence of resistance, high production costs and the fact that many AMP candidates do not progress successfully to clinical use [16]. This review summarises the main characteristics, mechanisms of action and applications of AMPs, bacteriocins and mycocins and discusses the key challenges that still limit their broader translation. This narrative review was based on a literature search conducted in PubMed and Google Scholar, complemented by manual screening of relevant references cited in selected articles, and focusing primarily on studies published within the last 10 years. Older studies were included when relevant to provide historical context, describe established mechanisms, or discuss antimicrobial peptides, bacteriocins or mycocins already used in clinical or applied settings. Search terms included combinations of “antimicrobial peptide”, “antimicrobial peptides”, “AMP”, “bacteriocin”, “mycocin”, “natural antimicrobial”, “food safety”, “agriculture”, “animal health”, “healthcare”, “antibiotic resistance”, “biofilm”, “toxicity”, “pharmacokinetics” and “pharmacodynamics”. Studies were included when they addressed antimicrobial activity, mechanisms of action, applications, translational potential or limitations of AMPs, bacteriocins, or mycocins in food safety, agriculture, animal health or healthcare. Studies were excluded when they were not directly related to antimicrobial or anti-infective applications, focused primarily on unrelated biological functions or were outside the scope of the review.

## 2. Natural Antimicrobials: AMPs, Bacteriocins and Mycocins

Natural antimicrobials comprise a chemically diverse group of compounds, including peptide- and protein-based molecules as well as non-peptide secondary metabolites such as polyphenols, terpenoids, alkaloids, organosulfur compounds and other plant- or bee-derived substances, including honey, propolis, garlic and onion derivatives [17,18,19,20]. These non-peptide antimicrobials have been extensively investigated in food preservation, agriculture and healthcare. However, a comprehensive discussion of all natural antimicrobial classes would require a broader chemical and regulatory framework than can be adequately covered here. Therefore, the present review deliberately focuses on peptide-based natural antimicrobials, namely AMPs, bacteriocins and mycocins. Non-peptide natural antimicrobials are mentioned only when useful as contextual benchmarks, but they are not the primary subject of this review.

### 2.1. AMPs

AMPs comprise a diverse group of naturally occurring or engineered antimicrobial molecules that play important roles in microbial competition and innate defence across bacteria, fungi, plants, animals and humans [21,22]. Although many classical AMPs are short peptides of approximately 12–50 amino acids, this definition is not absolute, as antimicrobial peptide-like molecules vary widely in size, net charge, hydrophobicity, secondary structure and biosynthetic origin. Most well-characterised AMPs are cationic and amphipathic, which favours their interaction with negatively charged microbial surfaces, but anionic, neutral, cyclic, lipidated and highly modified peptides have also been described [23,24]. Some AMPs contain disulphide bridges, D-amino acids, non-proteinogenic residues or post-translational modifications, while others, such as peptaibols, lipopeptides or lantibiotic-like molecules, can be considered non-classical antimicrobial peptide-based compounds. This structural and chemical diversity contributes to their broad range of mechanisms and applications. They are known for broad-spectrum activity against bacteria, fungi, viruses and parasites, largely due to their capacity to interact with microbial membranes and compromise membrane integrity [23]. Most AMPs are cationic and amphipathic, which favours their interaction with negatively charged microbial surfaces while reducing toxicity toward host cells [23]. However, not all AMPs are cationic. Anionic antimicrobial peptides (AAMPs), although less studied, also contribute to innate immunity in humans, animals and plants. These peptides, typically with net charges ranging from −1 to −7, possess amphiphilic properties and may act by forming salt bridges with microbial membranes or by targeting intracellular components [24]. Examples include dermcidin from human sweat and otacidin from amphibians [24,25,26,27].

AMPs can be categorized based on their structure. The main structural classes include the following: (1) α-helical peptides, like magainins and cecropins, which are flexible in solution but adopt helical shapes upon contact with membranes; (2) β-sheet peptides, such as defensins, stabilized by disulfide bonds; (3) extended peptides that are rich in specific amino acids like proline or tryptophan, such as indolicidin; and (4) loop or cyclic peptides, including bactenecins. These structural features are functionally relevant, as amphipathicity, charge distribution, cyclization, disulfide bonds and other conformational constraints can influence membrane interaction, resistance to proteolytic degradation, stability in complex matrices and overall antimicrobial activity.

Beyond structural classification, AMPs can also be categorized according to their mode of action. Many exert antimicrobial effects by forming pores or disrupting microbial membranes, whereas others enter the cell and affect essential intracellular processes, including DNA replication, RNA transcription and protein synthesis. In some cases, AMPs can also trigger programmed cell death-like responses in target microorganisms. This diversity in structure and function supports the exploration of AMPs as antimicrobial tools in food safety, agriculture and healthcare, but their suitability is compound- and application-specific and depends on stability, toxicity, antimicrobial spectrum, matrix compatibility, production feasibility and regulatory requirements.

#### 2.1.1. Non-Bacteriocin Bacterial-Derived AMPs

Although bacteriocins can be considered within the broader group of bacterial antimicrobial peptides and proteins, they are discussed separately in Section 2.3 because of their ribosomal biosynthesis, specific classification systems, regulatory relevance and well-established applications, particularly in food biopreservation. Therefore, this subsection focuses on non-bacteriocin bacterial-derived antimicrobial peptides and peptide-like compounds that contribute to microbial competition, niche colonization and defence against competing microorganisms. These include non-ribosomal peptides, cyclic peptides, lipopeptides and hybrid peptide-based secondary metabolites produced through specialised biosynthetic pathways [28,29,30]. Concrete examples include *Bacillus*-derived lipopeptides such as surfactins, iturins and fengycins, which are synthesized by non-ribosomal peptide synthetases and contribute to antimicrobial activity, biofilm modulation, plant-disease suppression and biofungicide development [31]. Their technological relevance is particularly evident in agricultural biocontrol, where they can inhibit phytopathogenic fungi and bacteria and support more sustainable crop-protection strategies. Another relevant example is daptomycin, a non-ribosomal cyclic lipopeptide produced by *Streptomyces roseosporus*, which illustrates the clinical potential of bacterial lipopeptides as membrane-active agents against resistant Gram-positive pathogens [32].

Owing to their structural diversity and activity against pathogenic and multidrug-resistant microorganisms, these bacterial-derived compounds have attracted interest as potential alternatives or adjuvants to conventional antibiotics. Depending on their structure and target organism, these molecules may display antibacterial, antifungal, antibiofilm or membrane-active properties [28,29,30].

The antimicrobial activity of these compounds is mediated by multiple and sometimes complementary mechanisms. Many interact with microbial membranes, causing pore formation, membrane destabilization, dissipation of membrane potential or leakage of intracellular components [23,33,34,35]. Others interfere with essential intracellular processes, including DNA, RNA or protein synthesis, enzyme activity, cell division or metabolic homeostasis. Some peptide-based compounds may also promote oxidative stress through reactive oxygen species (ROS) accumulation, contributing to microbial damage and cell death [23,30,33,35].

This mechanistic diversity supports the potential use of non-bacteriocin bacterial-derived AMPs and peptide-like compounds in food safety, agriculture, cosmetics and healthcare. However, their translation remains limited by susceptibility to proteolytic degradation, reduced stability under physiological or processing conditions, possible cytotoxicity or haemolytic activity, context-dependent antimicrobial spectra, high production costs and difficulties in scale-up and purification [28,29,30,36]. Production and downstream processing illustrate these limitations. For example, optimisation of medium composition and fermentation parameters increased surfactin production by *Bacillus subtilis* YPS-32 to 1.82 g/L, showing that yield can be improved through response-surface-based fermentation optimisation [37]. Similarly, *Bacillus velezensis* SK was reported to produce a lipopeptide mixture at 1.33 g/L, but purification required sequential extraction and chromatographic steps to resolve surfactin isoforms and related compounds [38]. These examples highlight a common challenge for bacterial lipopeptides: they are frequently produced as mixtures of homologues or co-produced families, which complicates purification, quantification and batch-to-batch standardisation. Clinically relevant lipopeptides face similar production challenges; for example, daptomycin production by *Streptomyces roseosporus* has been improved through metabolic engineering strategies targeting precursor supply, regulatory pathways, by-product formation, biosynthetic gene-cluster copy number and oxygen supply [39]. Therefore, optimisation of yield, product purity, congener composition and reproducible biological activity remains essential before broader technological application. Strategies such as peptide engineering, recombinant expression, synthetic biology, encapsulation, nanocarriers and optimized fermentation systems are being explored to improve stability, bioavailability, specificity and production yield [29,30,35,36].

#### 2.1.2. Filamentous Fungi-Derived AMPs

Filamentous fungi are prolific producers of structurally diverse antimicrobial peptides and peptide-like compounds, including peptaibols, peptaibiotics and other antifungal proteins with targeted antimicrobial activity. These molecules contribute to ecological competition, particularly in antagonistic interactions with other fungi, bacteria and plant pathogens, and have attracted interest for applications in agriculture, food safety and biotechnology.

Peptaibols are among the best-characterized antimicrobial peptides produced by filamentous fungi. They are linear peptides enriched in non-proteinogenic amino acids, particularly α-aminoisobutyric acid (Aib), which promotes the formation of stable helical structures and facilitates their insertion into lipid bilayers [40,41]. Once inserted into target membranes, peptaibols can form transmembrane pores, disturb ion gradients and compromise cellular homeostasis, ultimately leading to membrane permeabilization and cell death. Prominent examples include alamethicin, trichogin, suzukacillin and zervamicin [40,41]. These compounds are commonly associated with *Trichoderma* spp., which are widely studied for their antagonistic activity against phytopathogenic fungi, including *Botrytis cinerea* [40,41,42].

Other filamentous fungi also produce antimicrobial peptide-like compounds with distinct biological activities. Emericellipsin A, produced by *Emericellopsis alkalina*, has shown antifungal activity, while efrapeptins from *Tolypocladium niveum* act mainly by inhibiting mitochondrial ATP synthase and disrupting cellular energy metabolism [43,44,45]. Although cytotoxic, insecticidal or antiviral effects have also been reported for some of these compounds; these activities are outside the main scope of this review and are mentioned only briefly as examples of broader bioactivity [43,44,45,46]. Here, their relevance is considered primarily in relation to antifungal activity, food safety, post-harvest protection and agricultural biocontrol. The mechanisms of action of filamentous fungi-derived antimicrobial peptides and proteins vary according to compound family. Peptaibols primarily act through interactions with fungal membranes, leading to pore formation, ion leakage and loss of cellular homeostasis [40,41,42]. In contrast, small cysteine-rich antifungal proteins secreted by filamentous fungi may involve more complex cellular responses. For example, PAF and PAFB from *Penicillium chrysogenum* exhibit antifungal activity associated with fungal membrane lipid composition and regulated cellular uptake, suggesting mechanisms that are not restricted to canonical membrane permeabilisation [47].

PAF from *P. chrysogenum* has been shown to affect plasma membrane integrity and induce an apoptosis-like phenotype in *Aspergillus nidulans*, with associated oxidative stress responses [48,49]. In addition, PAFC from *P. chrysogenum* Q176 reduced the metabolic activity of pre-established *Candida albicans* biofilms, including a fluconazole-resistant clinical isolate, and its candidacidal activity was associated with intracellular ROS induction, protein internalisation and plasma membrane disintegration [50]. These findings support the potential of filamentous fungi-derived antimicrobial proteins as antifungal tools in food safety, agriculture and healthcare, while also highlighting the need for compound-specific mechanistic evaluation.

#### 2.1.3. Yeast-Derived AMPs

Yeasts are increasingly recognized as sources of antimicrobial molecules, including low-molecular-weight antimicrobial peptides, proteinaceous toxins and other secreted bioactive compounds that contribute to microbial competition and fermentation-associated microbial control. Although yeast killer toxins and mycocins are discussed separately in Section 2.2, several studies have reported peptide-like antimicrobial fractions produced by yeasts with activity against spoilage or pathogenic microorganisms.

This antimicrobial potential is particularly relevant in fermentation microbiology. In wine, bioethanol and fermented-food production, microbial contaminants can reduce product quality, alter sensory properties, decrease fermentation efficiency or cause spoilage [51]. Wine spoilage yeasts such as *Brettanomyces bruxellensis* are especially problematic because they can produce undesirable volatile phenols and persist under stressful fermentation and storage conditions [52]. In bioethanol fermentations, contaminating yeasts and bacteria may compete with starter strains, reduce ethanol yield and compromise process stability [53]. Yeast-derived AMPs and peptide fractions may therefore provide targeted biocontrol tools that are compatible with fermentation-based processes.

Non-*Saccharomyces* yeasts have attracted particular interest as sources of antifungal peptides for food and fermentation-related applications. For example, *Candida intermedia* LAMAP1790 has been reported to produce low-molecular-weight antimicrobial peptides (<10 kDa) with activity against the wine-spoilage yeast *Brettanomyces bruxellensis*, without markedly affecting fermentative *Saccharomyces cerevisiae* [54]. Subsequent work showed that peptide-containing fractions from this strain also affect other spoilage yeasts, including *Pichia guilliermondii*, and may induce reactive oxygen species accumulation in sensitive cells [55]. These findings support the potential of non-*Saccharomyces* yeasts as sources of selective antimicrobial peptides for biocontrol in fermented products.

In parallel, *S. cerevisiae* has been shown to secrete antimicrobial peptides during alcoholic fermentation. Studies on wine and industrial strains identified glyceraldehyde 3-phosphate dehydrogenase (GAPDH)-derived peptides with activity against wine spoilage yeasts and bacteria. GAPDH-derived antimicrobial peptides from *S. cerevisiae* have been detected in extracellular fractions during alcoholic fermentation and, in later studies, associated with the cell surface/cell wall of stationary-phase cells [56,57,58,59]. Their occurrence therefore appears to be strain-, growth-phase- and fermentation-condition-dependent, and their release should not be attributed simply to nonspecific cell lysis. Multiple studies by Branco and collaborators demonstrated that GAPDH-derived peptides from *S. cerevisiae*, named saccharomycin, inhibit the growth of contaminants such as *Brettanomyces bruxellensis*, a major spoilage yeast in bioethanol and wine fermentations [56,57,58,59,60,61]. For example, saccharomycin showed MIC values of 1–2 mg/mL against six *B. bruxellensis* strains, including ISA 1649, ISA 1700, ISA 1791, ISA 2104, ISA 2116 and ISA 2211 [62]. In wine-related conditions, 1.0 mg/mL saccharomycin combined with 25 mg/L SO_2_ prevented *B. bruxellensis* proliferation in wines containing 13–14% ethanol, supporting its potential as a biocontrol strategy to reduce reliance on high SO_2_ levels [62]. The antimicrobial activity of peptide fractions secreted by *S. cerevisiae* has also been quantified against foodborne and clinically relevant microorganisms. A 2–10 kDa peptide fraction derived from *S. cerevisiae* metabolism showed MIC values of 0.25 mg/mL against *Escherichia coli*, *Listeria monocytogenes* and *Salmonella* sp., whereas the MIC against *Candida albicans* was 1.0 mg/mL and *Candida krusei* was not inhibited at the highest concentration tested [61]. These values indicate that the activity of yeast-derived peptide fractions is strongly dependent on the target microorganism and cannot be generalized without specifying the active concentration and assay conditions.

Genetic engineering strategies have been employed to enhance the production of these AMPs in industrial strains. Overexpression of GAPDH fragments, particularly partial *TDH1* gene sequences in *S. cerevisiae*, led to significant inhibition of *B. bruxellensis*—up to 72-fold greater than that observed with unmodified strains—while maintaining ethanol production efficiency. This suggests that AMP-overproducing yeast strains could be used to naturally control microbial contamination, reducing reliance on chemical antimicrobials in industrial processes [63]. In this case, the biological effect was reported as relative inhibition by AMP-overproducing yeast strains rather than as a purified peptide MIC.

Beyond fermentation, yeast-derived peptide fractions have also been explored for cosmetic and dermocosmetic applications, particularly as multifunctional antimicrobial and skin-related bioactives [64]. These applications, including reported MIC values, preservative efficacy and skin-related bioactivities, are discussed in more detail in Section 3.2.

### 2.2. Mycocins

Mycocins, also known as yeast killer toxins, should be distinguished from broader yeast-derived AMP fractions. Whereas yeast-derived AMPs often refer to low-molecular-weight peptide fractions or peptide-like compounds with antimicrobial activity, mycocins are typically extracellular proteins or glycoproteins produced by killer yeasts. They are associated with the killer phenotype, in which a producer strain secretes a toxin capable of inhibiting or killing susceptible microorganisms while remaining immune to its own toxin. This toxin–immunity relationship distinguishes mycocins from many general antimicrobial peptide fractions and contributes to their ecological role in microbial competition [65,66]. This specificity, together with their proteinaceous nature and low reported toxicity in several experimental models, makes them interesting candidates for food biopreservation, biotechnology and biomedical antifungal applications, although safety and efficacy must be assessed case by case.

The genetic determinants of mycocin production are diverse. In *S. cerevisiae*, the best-characterized killer systems are associated with cytoplasmic double-stranded RNA viruses involving a helper L-A virus and a satellite M virus. The M dsRNA encodes specific killer toxins, such as K1, K2, K28 or Klus, as well as the corresponding immunity determinant [66]. These toxins act through distinct mechanisms, including binding to β-1,6-glucans in the fungal cell wall, disruption of plasma membrane function or interference with DNA synthesis and cell cycle regulation [66]. However, killer phenotypes are not restricted to dsRNA viral systems. In other yeasts, they may be associated with linear dsDNA plasmids, virus-like elements or chromosomal determinants. Thus, mycocins represent a heterogeneous group of yeast-derived antimicrobial proteins with different genetic origins, molecular structures, target spectra and mechanisms of action.

Antibacterial effects have also been reported for some yeast extracellular preparations, particularly from *Wickerhamomyces anomalus*, but these findings should be interpreted cautiously. Calazans et al. evaluated the antimicrobial activity of culture supernatants from *W. anomalus* WA45 against 29 coagulase-positive *S. aureus* strains isolated from beef, pork and chicken, together with one reference strain [67]. In that study, the active extracellular preparation was described as containing mycocins, and its activity was expressed in β-glucanase units. Complete inhibition of the tested *S. aureus* strains was observed at higher β-glucanase activities, while inhibition was still detected at 0.02 U/mg [67]. Therefore, the reported antibacterial effect should be considered the activity of an extracellular mycocin/β-glucanase-containing preparation rather than the action of a single purified and structurally defined mycocin. Further purification, molecular identification and mechanistic studies are required to determine whether the antibacterial activity is mediated by a specific mycocin, extracellular enzymes with antimicrobial effects, other extracellular factors, or a combination of these components [67].

One of the noteworthy mycocin-producing genera is *Debaryomyces*, particularly the species *Debaryomyces hansenii.* This yeast is commonly found in cheeses and other fermented foods, where it survives under high-salt, low-pH, and low-water-activity conditions, making it well-suited for food preservation applications. Banjara et al. (2016) screened 42 isolates of *D. hansenii* from 22 cheese samples and found that 23 strains exhibited killer activity against both *Candida albicans* and *Candida tropicalis* [67,68]. These observations suggest that *D. hansenii* may contribute to the regulation of fungal communities in fermented food matrices, although any potential role in the human gastrointestinal microbiome would require dedicated in vivo validation. *D. hansenii* is included in the EFSA list of microorganisms with Qualified Presumption of Safety (QPS) status, supporting its relevance for food-and feed-related applications when used under appropriate conditions [69]. However, QPS status applies to the taxonomic unit and does not automatically establish the safety or regulatory acceptability of every strain, mycocin preparation, extract or specific application. Therefore, strain-level safety assessment and application-specific validation remain necessary before use in fermented-food bioprotection. Practical application as a mycocin-based preservative will also require further characterisation of the active molecule, production yield, stability, effective concentration in food matrices and safety under realistic exposure conditions.

Beyond food safety, mycocins have also been discussed as potential antimicrobial tools for health-related applications. However, the available studies involve different yeast species, toxin preparations, target organisms and assay formats, and should therefore be interpreted in relation to the specific mycocin, active concentration or activity unit, target organism and experimental model used [65]. Broad claims regarding antibacterial or antiprotozoal activity should be avoided unless they are supported by purified compounds, defined concentrations and appropriate mechanistic evidence.

Additionally, Golubev et al. demonstrated mycocin production in *Trichosporon pullulans* isolates obtained from tree exudates released during early spring [70].

In that study, mycocinogenic activity was assessed using agar-based inhibition and culture-supernatant assays rather than a purified mycocin preparation; therefore, no MIC or defined mycocin concentration was reported. Mycocinogenic activity was associated with dsRNA-containing virus-like particles and conferred a competitive advantage over sensitive strains in mixed-culture experiments and natural populations. The mycocins displayed a very narrow activity spectrum, inhibiting only conspecific isolates from tree exudates, with no detectable activity against the other yeast species tested [70].

Overall, mycocins represent a versatile group of yeast-derived antimicrobial molecules with potential applications in food biopreservation, biotechnology and antimicrobial therapy. However, their broader use will require further characterisation of spectrum of activity, stability, production feasibility, safety and performance in realistic matrices.

### 2.3. Bacteriocins

Bacteriocins are ribosomally synthesised antimicrobial peptides or proteins produced by bacteria and are among the best-characterised groups of bacterial antimicrobial compounds. Unlike many broad-spectrum antimicrobial agents, bacteriocins often display narrow or target-specific activity, frequently against closely related species or selected pathogens [71]. This specificity, together with their proteinaceous nature, biodegradability and generally low toxicity toward eukaryotic cells, has supported their development as natural antimicrobial tools for food preservation, healthcare, veterinary applications and biotechnology [36].

Bacteriocin production is especially well documented in lactic acid bacteria (LAB), where these molecules contribute to ecological competitiveness in fermented foods and gastrointestinal or environmental niches. LAB-derived bacteriocins can inhibit relevant foodborne and spoilage microorganisms, including *Listeria monocytogenes* and *Staphylococcus aureus*, making them particularly attractive for food biopreservation and multi-hurdle preservation strategies [72]. However, bacteriocins are not restricted to LAB and have also been described in other Gram-positive and Gram-negative bacteria, expanding their structural and functional diversity.

Structurally and biosynthetically, bacteriocins from Gram-positive bacteria have been classified using several schemes, and these systems have evolved as new molecules and biosynthetic pathways have been described. Although no single classification is universally adopted, commonly used frameworks distinguish post-translationally modified peptides, such as lantibiotics—small, heat-stable, non-lanthionine peptides, such as pediocin-like bacteriocins, enterocins and plantaricins—and larger, heat-labile protein bacteriocins with bacteriolytic or non-lytic mechanisms [71,73]. Class I bacteriocins, or lantibiotics, are small post-translationally modified peptides containing unusual amino acids such as lanthionine; nisin and lacticin 3147 are prototypical examples. Class II bacteriocins are small, heat-stable, non-lanthionine peptides, including pediocin PA-1, enterocins and plantaricins, many of which show strong activity against *Listeria* spp. Class III bacteriocins are larger, heat-labile proteins with bacteriolytic or non-lytic mechanisms [12,71,73].

Older schemes also included a class IV category for complex bacteriocins associated with lipid or carbohydrate moieties. However, this category remains debated, because in many cases the contribution of the non-protein component to antimicrobial activity has not been clearly demonstrated or the compounds were not sufficiently purified and chemically characterized. Therefore, class IV should be interpreted cautiously, and bacteriocin classification should be viewed as an evolving framework based on biosynthesis, structure, post-translational modification, molecular size and genetic organization [12,71,73].

The mechanisms of action of bacteriocins are diverse but commonly involve disruption of membrane integrity, receptor-mediated pore formation or interference with cell wall biosynthesis. Lantibiotics such as nisin bind lipid II, thereby inhibiting peptidoglycan biosynthesis and promoting pore formation in the cytoplasmic membrane, which leads to dissipation of membrane potential and leakage of essential ions and metabolites [74]. Many class II bacteriocins act after receptor recognition at the target cell surface, inserting into the membrane and disturbing membrane organization. Other bacteriocins may affect intracellular targets, including DNA, RNA or protein synthesis, although these mechanisms are less common and often depend on the specific bacteriocin and target organism [36,71].

Among LAB-derived bacteriocins, nisin remains the best-studied and most widely applied example. Produced by *Lactococcus lactis*, nisin has GRAS status and is authorised in numerous countries for use in dairy products, canned foods and other food matrices. Its dual mechanism, involving lipid II binding and membrane pore formation, explains its strong activity against Gram-positive bacteria, including *L. monocytogenes* and spore-forming bacteria. Beyond food preservation, nisin has also been evaluated in pharmaceutical, oral-care, wound-care and cosmetic contexts as a natural antimicrobial component [75,76,77,78]. However, despite its established use, nisin activity can be reduced in complex matrices because of adsorption to proteins or fat components, degradation by proteolytic enzymes, pH- or salt-dependent effects and limited diffusion or uneven distribution in the final product. In addition, tolerance or resistance to nisin may develop in target microorganisms through changes in cell envelope composition, membrane charge, stress-response pathways, target accessibility or proteolytic inactivation. Therefore, nisin is often more effective when used within multi-hurdle preservation strategies or delivery systems that improve stability, availability and local antimicrobial activity [79,80,81].

Other bacteriocins, including pediocin PA-1 from *Pediococcus acidilactici*, enterocins from *Enterococcus faecium* and plantaricins from *Lactiplantibacillus plantarum*, (formerly known as *Lactobacillus plantarum*) have shown potential for dairy preservation, control of *L. monocytogenes* in cheeses and fermented products and use as adjuncts in multi-hurdle preservation systems [71]. Bacteriocin-like inhibitory substances (BLIS) from non-LAB genera, including *Bacillus* and *Staphylococcus*, further expand the available antimicrobial repertoire, with activity against diverse Gram-positive and, in some cases, Gram-negative bacteria, particularly when combined with permeabilising agents or other hurdles [82].

A major limitation of bacteriocin application is the possible loss of activity in complex matrices. Bacteriocins may be degraded by proteases, adsorb to food components, interact with proteins or lipids, or show reduced activity under unfavourable pH, salt or temperature conditions. Therefore, recent research has focused on formulation and delivery strategies to improve bacteriocin stability, bioavailability and controlled release [83,84]. Encapsulation in liposomes, polymeric micro- and nanoparticles, edible films or nanovesicles can protect bacteriocins from degradation, reduce interactions with food matrices and enhance antimicrobial efficacy during storage. For example, liposomal or nanovesicle formulations of nisin and other bacteriocins in dairy systems have improved anti-*Listeria* activity and allowed lower effective doses, thereby reducing potential sensory impacts [85].

Food-matrix applications further illustrate the technological relevance of bacteriocins. In dairy products, nisin and other LAB-derived bacteriocins have been widely investigated for the control of *Listeria monocytogenes*, *Staphylococcus aureus* and spore-forming bacteria in cheese, pasteurized cheese spreads and fermented dairy products [86,87]. In meat and poultry products, bacteriocins such as nisin and pediocin-like peptides have been evaluated to reduce *L. monocytogenes* and other spoilage or pathogenic bacteria, particularly when combined with refrigeration, modified-atmosphere packaging, organic acids, high-pressure processing or other hurdle technologies [88,89]. In beverages and acidic food systems, bacteriocins may help control spoilage bacteria or sensitive Gram-positive contaminants, although efficacy depends strongly on pH, ionic strength, processing conditions and interactions with matrix components [78,83,84]. Active packaging represents another promising approach: incorporation of bacteriocins into edible films, coatings, cellulose-based materials or polymeric packaging can provide localized antimicrobial activity at the food surface, where post-processing contamination frequently occurs [89,90]. These examples show that bacteriocins are not universally effective across all matrices, but can be valuable when formulation, delivery strategy and target food system are matched appropriately [83,84,85].

Beyond food preservation, bacteriocins are increasingly recognised as potential therapeutic agents, topical antimicrobials, disinfectants and microbiome-modulating tools [91]. Their relatively narrow spectrum may reduce collateral damage to beneficial microbiota, while their proteinaceous nature favours environmental degradation compared with persistent broad-spectrum biocides [36]. Nevertheless, their successful translation requires careful evaluation of stability, safety, resistance development, production scalability and efficacy in realistic matrices.

**Figure 1:**
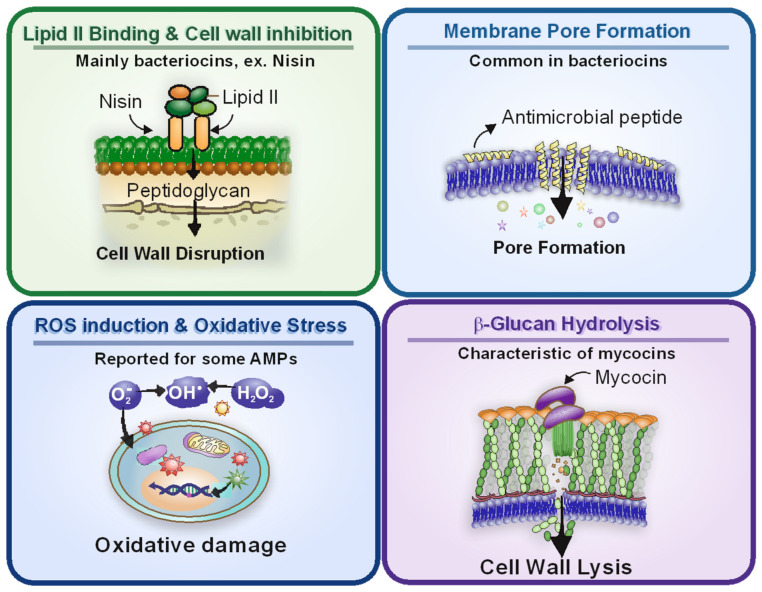
Representative mechanisms of action of antimicrobial peptides (AMPs), bacteriocins and mycocins. AMPs, bacteriocins and mycocins may exert antimicrobial activity through distinct but sometimes overlapping mechanisms. Many AMPs disrupt microbial membranes by increasing membrane permeability or promoting pore formation. Lantibiotic bacteriocins, such as nisin, bind lipid II, inhibiting peptidoglycan biosynthesis and, in some cases, promoting pore formation in the cytoplasmic membrane. Yeast-derived mycocins may act on fungal cell wall or membrane targets, depending on the producing species and toxin type. In some cases, their activity has been associated with β-glucan hydrolysis, which can compromise fungal cell wall integrity and lead to cell damage or lysis. Antimicrobial activity may also involve reactive oxygen species (ROS) generation, resulting in oxidative damage to cellular components. Multimodal mechanisms of action may help limit the emergence of microbial resistance. Arrows indicate the direction of antimicrobial action or downstream cellular effects, while graphical symbols represent the specific mechanisms shown in each panel.

The main antimicrobial mechanisms described for AMPs, bacteriocins and mycocins are summarised in Figure 1.

## 3. Applications of Natural Antimicrobials as Preservatives

### 3.1. Potential as Natural Preservatives for Food Products

Bacteriocins, yeast-derived mycocins and other antimicrobial peptide-like compounds are promising natural preservation tools, as their proteinaceous nature and activity against spoilage and pathogenic microorganisms make them relevant for improving microbial safety and extending food shelf life [92].

Bacteriocins are ribosomally synthesized antimicrobial peptides produced by various bacteria, particularly LAB, that show potent inhibitory effects against foodborne pathogens and spoilage organisms [93]. Among these, nisin, produced by *L. lactis*, is the most commercially successful bacteriocin and has been widely applied in food preservation systems.

Nisin is typically produced through fermentation using milk-based substrates, after which it is concentrated and lyophilized for use in various food matrices. It is particularly effective against Gram-positive bacteria, such as *L. monocytogenes* and *Clostridium botulinum*, and is approved for use in dairy products, canned foods, and cured meats [94] In food challenge studies, its efficacy is clearly concentration dependent. For example, nisin treatments at 25 and 250 ppm were evaluated against *L. monocytogenes* inoculated on cold-smoked salmon at approximately 10^2^ CFU/g and stored at 4 or 7 °C; the 250 ppm treatment showed greater efficacy than 25 ppm and reduced the prevalence of *L. monocytogenes* in treated samples [94]. Pediocin PA-1 also displays strong anti-*Listeria* activity, with synthetic pediocin PA-1 showing a MIC of 6.8 nM against *L. monocytogenes* [95,96].

Despite its broad utility, nisin has several application-dependent limitations. Its thermal stability is pH- and process-dependent, with better retention of activity under acidic conditions and greater loss under neutral or alkaline pH and prolonged heating. It is also susceptible to degradation by proteolytic enzymes, including proteinase K, trypsin and chymotrypsin, and its activity may be reduced by interactions with food-matrix components such as proteins, lipids and salts [97,98]. Therefore, nisin and other bacteriocins are often more effective when incorporated into multi-hurdle preservation strategies combining refrigeration, pH control, organic acids, modified-atmosphere packaging, high-pressure processing, permeabilising agents or encapsulation systems [36,99,100].

In purified peptide assays, synthetic pediocin PA-1 showed anti-*Listeria* activity in the nanomolar range, with a reported MIC of 6.8 nM against *L. monocytogenes* [95]. Mechanistic studies also show that nisin dissipated the proton motive force of *L. monocytogenes* Scott A at concentrations ≥ 5 µg/mL, whereas 1 µg/mL mainly affected the pH gradient and had limited effect on membrane potential [101]. Similarly, pediocin PA-1 at 20 µg/mL caused marked or complete dissipation of the proton motive force in energized *L. monocytogenes* Scott A cells [102]. These examples show that bacteriocin efficacy should not be generalized without specifying concentration, target organism, physiological state, assay system and matrix conditions. Although the present review focuses mainly on AMPs, bacteriocins and mycocins, other microbial natural antimicrobials, such as natamycin, provide useful benchmarks for food-preservation applications. Natamycin is not an AMP, bacteriocin or mycocin, but a polyene macrolide antifungal compound produced by *Streptomyces natalensis* [103]. Its inclusion here is therefore limited to its role as a non-peptide comparator for established antifungal preservation strategies, particularly in surface treatment of cheese and dry or cured meat products. Unlike many peptide-based antimicrobials that act through membrane disruption or pore formation, natamycin acts mainly by binding ergosterol in fungal membranes and inhibiting fungal growth without classical membrane permeabilisation [104]. Regulatory authorities, including the EFSA and FDA, permit natamycin use as a food preservative under defined conditions, particularly for surface treatment of selected foods [104,105,106]. Because of its low water solubility, natamycin remains mainly at the food surface, making it suitable for controlling yeasts and moulds in cheese, dry or fermented meat products, bakery products, fruit and vegetable coatings and fermented foods such as olives [65,105,106,107,108,109,110,111,112,113]. More recently, natamycin has also been incorporated into biodegradable films and edible coatings based on materials such as starch, whey protein or chitosan, illustrating how active packaging can combine a physical barrier with localised antifungal activity [112]. This benchmark is useful because it illustrates the regulatory maturity, formulation control and matrix-specific validation that peptide-based antimicrobials would also need to achieve for broader application in food systems.

#### Synthetic and Engineered Peptide Analogues Inspired by Natural Antifungal Peptides

Synthetic and engineered peptide analogues should be distinguished from naturally occurring AMPs, bacteriocins and mycocins. They are not natural antimicrobials in the strict sense, but they are relevant to this review because many are designed from natural peptide scaffolds or inspired by naturally occurring antimicrobial motifs. These compounds illustrate how the structural principles of natural AMPs can be used to improve antifungal potency, stability, spectrum of activity and performance in food matrices.

Several synthetic peptides have demonstrated antifungal activity against common spoilage yeasts, such as *Zygosaccharomyces bailii*, *S. cerevisiae* and *D. hansenii* [114,115]. For instance, the synthetic peptide KKFFRAWWAPRFLK-NH_2_ and related analogues inhibited food-spoilage yeasts with MICs ranging from 25 to 200 µg/mL; *Z. bailii* was reported as the most sensitive species, whereas *Zygosaccharomyces rouxii* was the most resistant. These peptides acted through membrane permeabilization and maintained activity under varying pH, salt and thermal conditions, including application with experimental validation, was also reported in an orange soft drink model [115].

Similarly, the α-helical peptide SnuCalCpI15 from *Calotropis procera* bound to the cell surface and penetrated food spoilage yeasts, causing increased membrane permeability and structural damage to the cell wall [116]. In that study, SnuCalCpI15 showed MIC values of 0.20 ± 0.01 mM against *Candida albicans* and *Saccharomyces cerevisiae*, and 0.26 ± 0.01 mM against *Pichia anomala* and *Rhodotorula mucilaginosa* [116].

Other studies have evaluated barley-derived synthetic defensins and radish antimicrobial peptides. The barley-derived synthetic defensin D-lp1 showed MIC/MFC values of 50–100 µg/mL against *Z. bailii* and *D. hansenii*, while *S. cerevisiae* and *Z. rouxii* were inhibited only at higher concentrations, with MIC/MFC values in the 200–400 µg/mL range. D-lp1 also induced dose-dependent membrane permeabilization and ROS production at 100, 200 and 400 µg/mL, and retained inhibitory activity against *Z. bailii* in apple juice at 100–400 µg/mL [117,118]. Chemically synthesized radish peptides Rs-AFP1 and Rs-AFP2 were also reported to inhibit food-spoilage yeasts, with Rs-AFP2 associated with both membrane permeabilization and ROS overproduction, whereas Rs-AFP1 mainly induced ROS overproduction [118]. These peptide systems were evaluated in food-relevant matrices, including soft drinks, salad dressings and fruit juice models, and showed no detectable cytotoxicity or haemolytic activity under the experimental conditions tested [117,118].

Overall, these studies show that synthetic and engineered antifungal peptide analogues may be useful complementary tools for food preservation. However, they should be presented as peptide-based translational derivatives inspired by natural antimicrobial scaffolds, rather than as natural antimicrobials themselves. Broader application will require further validation of safety, sensory impact, regulatory status, production cost, stability and efficacy in realistic food matrices [119,120].

### 3.2. Potential as Natural Preservatives for Cosmetics

A clear regulatory distinction is needed when discussing peptide-based antimicrobials in cosmetic applications. In cosmetic products, antimicrobial preservatives are primarily intended to protect the formulation from microbial contamination during manufacturing, storage and consumer use, and in the EU permitted preservatives are listed under Annex V of Regulation (EC) No. 1223/2009 [121]. Their efficacy is commonly evaluated through preservative-efficacy or challenge tests, such as ISO 11930, which assess the antimicrobial protection of the cosmetic product rather than therapeutic activity on the skin [122]. By contrast, peptide-based ingredients investigated for antioxidant, anti-collagenase, soothing, barrier-supporting or skin-conditioning effects should be described as cosmetic or dermocosmetic actives only when the claims remain within the cosmetic framework and are supported by appropriate evidence, in accordance with the common criteria for cosmetic claims [123]. Claims implying treatment or prevention of infection, wound healing, pathological inflammation, acne as a disease, dermatitis or other skin disorders may fall outside the cosmetic category and require medicinal or other regulatory assessment [123]. Therefore, AMPs, bacteriocins, mycocins and yeast-derived peptide fractions should be discussed separately as potential formulation preservatives, cosmetic bioactives or therapeutic candidates, according to the intended use, evidence level and regulatory pathway.

The pharmaceutical and cosmetic industries have shown growing interest in natural AMPs and bacteriocins as multifunctional ingredients that combine antimicrobial preservation with additional skin benefits [20,124]. These molecules can serve as alternatives or complements to conventional synthetic preservatives, responding to consumer demand for “clean-label” and sustainable formulations, while contributing to product safety and stability [20,63]. In topical products, AMPs and bacteriocins may act both as in-package preservatives and as on-skin antimicrobial agents that modulate the cutaneous microbiota without the broad collateral impact associated with traditional biocides [125].

Maurício et al. assessed its preservative efficacy in cosmetic and topical formulations using in vitro susceptibility assays and ISO 11930 challenge testing [124]. Nisin alone inhibited Gram-positive microorganisms, particularly *Staphylococcus aureus* and *Bacillus* sp., but it was not sufficient to ensure broad preservative efficacy against all tested cosmetic contaminants [124]. Adequate preservative performance according to criterion A of ISO 11930 was achieved only when nisin was combined with EDTA and conventional preservatives. Specifically, the combination of 125 ppm nisin, 0.1% EDTA and 0.35% synthetic preservative system showed antimicrobial activity compliant with ISO 11930 criterion A [124]. These findings indicate that, in cosmetic formulations, nisin may be useful as part of a hurdle-preservation strategy that reduces reliance on higher concentrations of conventional synthetic preservatives rather than as a stand-alone broad-spectrum preservative [124]. This example also illustrates a broader formulation challenge: antimicrobial activity in vitro does not necessarily translate into sufficient preservative efficacy in the final cosmetic product. Interactions with surfactants, polymers, oils, proteins, salts, pH conditions or packaging materials may reduce peptide availability and antimicrobial performance. Therefore, peptide-based antimicrobials may need to be combined with approved preservatives, preservative boosters, chelating agents, encapsulation systems or other formulation strategies to meet preservative-efficacy requirements, such as those evaluated by ISO 11930. Yeast-derived peptide fractions have recently gained attention as multifunctional dermocosmetic actives. In a recent study, peptides in the 2–10 kDa range secreted by *S. cerevisiae* Ethanol-Red were evaluated for cosmetic applications and showed antimicrobial, anti-collagenase, anti-inflammatory, antioxidant and wound-healing-related activities. Their antimicrobial activity was concentration-dependent, with MICs of 125 µg/mL against *Staphylococcus aureus*; 250 µg/mL against *Bacillus cereus*, *Staphylococcus epidermidis*, *Escherichia coli* and *Pseudomonas aeruginosa*; 500 µg/mL against methicillin-resistant *S. aureus* (MRSA), *Enterococcus faecalis* and *Streptococcus mitis*; and 1000 µg/mL against *Candida albicans* and *Streptococcus pyogenes* [64]. In challenge tests, the peptide fraction reduced microbial counts according to ISO 11930:2019 criteria for several tested microorganisms, although it was not effective alone against *Aspergillus brasiliensis*. Synergy was observed when the peptide fraction was combined with a reduced concentration of a conventional preservative system, particularly against *C. albicans* and *A. brasiliensis* [64]. The same peptide fraction inhibited collagenase activity by 41.8%, 81.9% and 94.5% at 250, 500 and 1000 µg/mL, respectively, showed no significant cytotoxicity in dermal cells up to at least 500 µg/mL and was tested at 250 µg/mL in anti-inflammatory and wound-healing-related assays [64]. These features support its potential as a multifunctional dermocosmetic bioactive, although further formulation, safety and preservative-efficacy validation is required before broader cosmetic application. In addition, these peptides have been shown to downregulate pro-inflammatory cytokine expression in dermal cells, inhibit collagen-degrading enzymes, and promote cell migration, highlighting their potential as anti-ageing, soothing, and reparative ingredients for skincare formulations that also require effective microbial protection [64].

AMPs and bacteriocins can be formulated into various cosmetic delivery systems, including emulsions, hydrogels, micellar solutions and advanced nano-carriers [83]. Encapsulation in liposomes, polymeric nanoparticles or biopolymer-based films improves peptide stability against oxidation and proteolysis, minimises interactions with other formulation components and enables controlled release onto the skin surface [126]. Delivery systems developed for food applications may provide useful technological inspiration for cosmetic formulations, but their use in skincare products requires cosmetic-specific validation of compatibility, peptide stability, release profile, preservative efficacy, skin tolerance, sensory properties and regulatory suitability [127]. Beyond preservation inside the package, natural antimicrobials show promise as cosmetic bioactives and adjuvants in managing microbiota-associated skin conditions [128].

Because many AMPs also exhibit immunomodulatory effects, including reduction of pro-inflammatory mediators and support of barrier function, they may help restore microbiome balance rather than indiscriminately eradicating microorganisms, a key distinction from classical antiseptics [125,129,130].

Despite these advantages, several formulation and regulatory challenges still limit the widespread use of AMPs and bacteriocins as cosmetic preservatives and biocides. Peptide stability, potential interactions with surfactants or charged polymers and cost-effective large-scale production must be optimised to ensure robust performance across diverse product types [83,120,123,124,125].

Ongoing advances in peptide engineering, recombinant production and encapsulation technologies are expected to mitigate these barriers and facilitate the integration of AMPs, bacteriocins and yeast-derived peptides as next-generation natural preservatives and functional ingredients in skincare products [36].

## 4. Potential of Natural Antimicrobials in Healthcare

Natural antimicrobials, particularly AMPs, are increasingly being explored for healthcare applications, including their use as antibiotic alternatives, antibiotic adjuvants, topical antimicrobials, antibiofilm agents and modulators or biomarkers of host defence. However, the therapeutic potential of AMPs in healthcare should be interpreted cautiously, as successful translation depends not only on antimicrobial potency but also on safety, stability, delivery, manufacturability, resistance risk and efficacy in clinically relevant infection models. A search was performed in ClinicalTrials.gov using the exact phrase “antimicrobial peptides” in quotation marks in the “Other terms” field (https://clinicaltrials.gov/expert-search, accessed on 20 October 2025), retrieving 182 studies. For the purposes of this narrative review, the subsequent discussion focused on studies registered within the last 10 years and directly related to the antimicrobial or anti-infective relevance of AMPs. Studies were considered relevant when the AMP-related intervention, target or biological rationale was associated with antimicrobial activity, anti-infective applications or infection-related clinical contexts. Studies primarily focused on non-antimicrobial functions, such as the hormonal role of liver-expressed antimicrobial peptide 2 (LEAP-2), were excluded.

A detailed list of clinical trials involving colistin, C16G2 other antimicrobial peptide candidates and endogenous AMP biomarkers is provided in Supplementary Tables S1–S5. In the main text, representative examples are discussed to highlight the main translational trends.

### 4.1. Potential to Combat Antibiotic-Resistant Infections

Antimicrobial resistance (AMR) is one of the major biomedical and societal challenges of the 21st century. The overuse and misuse of antibiotics in humans, animals and plants have contributed to the spread of resistant pathogens, making infections harder to treat and increasing healthcare and economic burdens [4,131]. In parallel, antibiotic discovery continues to face major obstacles, including the repeated rediscovery of known molecules, difficulties in cultivating potentially useful microorganisms and the high cost and risk associated with the development of new antibiotics [131,132]. In this context, natural antimicrobials, particularly AMPs, are being explored as therapeutic candidates and antibiotic adjuvants because of their diverse mechanisms of action, including membrane disruption, pore formation, intracellular targeting, antibiofilm effects and immunomodulatory activity [133]. Representative clinical and translational examples are summarized in Table 1.

Recent studies focused on the discovery or design of AMPs with activity against resistant microorganisms, including the isolation of new peptide scaffolds from microbial or insect sources, the repurposing of phage-encoded peptides and the design of lipopeptide analogues with activity against multidrug-resistant isolates [134,135,136,137]. These approaches presented activity against clinically relevant pathogens, including *S. aureus*, *P. aeruginosa*, *K. pneumoniae*, *E. coli*, *Salmonella* and *Campylobacter* spp., including antibiofilm effects and susceptibility to proteolytic degradation [134,135,136,137]. Together, these studies highlight the potential of AMPs as antimicrobial agents, while also revealing the need to evaluate stability, spectrum of activity and efficacy in infection-relevant models.

The use of AMPs as antibiotic adjuvants is also gaining attention. Recent examples include peptides able to resensitize methicilin-resistant *Staphylococcus aureus* (MRSA), enhance the effect of ciprofloxaxin against *E. coli*, *K. pneumoniae*, *P. aeruginosa* and *Acinetobacter baumannii*, or enhance tetracycline efficacy in experimental infection models [138,139,140]. Together, these findings suggest that AMP-antibiotic combinations help restore the activity of conventional antibiotics, reduce effective drug concentrations and limit resistance development [138,139,140]. However, translation of these combinations will require carefull assessment of toxicity, pharmacokinetics/pharmacondynamics, dosing compatibility and efficacy in clinically relevant infection settings [16]. More complex delivery strategies are also being explored. Gasanov et al. (2025) engineered Wharton’s jelly-derived mesenchymal stem cells to express the AMP SE-33 and evaluated their safety and antimicrobial activity in a murine model of *S. aureus*-induced pneumonia [141,142]. The modified cells showed antimicrobial activity, promoted bacterial clearance from the lungs and attenuated inflammatory damage, while preclinical safety analyses indicated minimal toxicity and immunogenicity [141,142]. Although still experimental, this approach illustrates the potential of cell-based platforms for localized or sustained AMP delivery.

Among clinically used AMP-related compounds, colistin and polymyxin B remain important examples because of their role in the treatment of severe infections caused by multidrug-resistant Gram-negative bacteria. Although colistin and polymyxin B are AMP-related cyclic lipopeptides of bacterial origin, they should be distinguished from newly developed natural or engineered AMP candidates. In this review, they are included as clinically approved benchmark examples of AMP-related compounds, rather than as newly developed AMP therapeutics, since they are long-established conventional last-resort antibiotics whose use is mainly associated with severe multidrug-resistant Gram-negative infections and is limited by toxicity, pharmacokinetics/pharmacodynamics uncertainty and resistance concerns. Polymyxins are cyclic lipopeptide AMPs produced by *Paenibacillus polymyxa* subsp. *colistinus*, with polymyxin B and polymyxin E, also known as colistin, being the clinically relevant members [143,144]. Colistin is available as colistin sulfate for oral or topical administration and as colistin methanesulfonate, an intravenous or nebulized prodrug [143,144]. Although colistin was approved by the FDA in 1959, its use declined because of nephrotoxicity and neurotoxicity, before re-emerging as a last-resort option against multidrug-resistant and extensively drug-resistant Gram-negative pathogens [143,144]. However, clinical trials have shown limited efficacy advantages for some combination regimens, relevant safety concerns and persistent pharmacokinetic and pharmacodynamic uncertainties, including optimal dosing, tissue penetration and conversion of colistin methanesulfonate to active colistin [145,146,147,148,149,150,151,152,153,154,155,156,157,158,159,160]. A detailed list of clinical trials involving colistin and polymyxin-based therapies is provided in Supplementary Table S1.

Targeted AMPs have also reached clinical evaluation. C16G2 is a specifically targeted antimicrobial peptide (STAMP) designed by fusing a broad-spectrum AMP domain with a *Streptococcus mutans*-specific targeting domain, enabling selective killing of *S. mutans* while preserving the surrounding oral microbiota [161]. Thus, C16G2 represents a clinically relevant example of a targeted, microbiome-sparing AMP strategy. Clinical studies support the safety, specificity and microbiome-sparing potential of C16G2 as a preventive strategy for dental caries, although broader clinical translation still requires further validation of long-term efficacy and implementation feasibility. Details of the C16G2 clinical trials are provided in Supplementary Table S2.

Other AMP candidates are being developed for topical, oral, wound-care and mucosal applications. Among clinical-stage candidates, TAPS-18, a synthetic cathelicidin-based peptide formulated as a topical gel, has been evaluated as an adjunct to non-surgical periodontal therapy (NCT05125718). PL-5, also known as peceleganan, is a hybrid cecropin–melittin-derived peptide under clinical evaluation as a topical spray for infected diabetic wounds (NCT06189638). PL-18, a synthetic AMP formulation administered as vaginal suppositories, is being assessed in a Phase I clinical trial for bacterial vaginosis and mixed vaginal infections, with attention to safety, tolerability and microbiota preservation (NCT05340790). In contrast, nisin-based formulations are mainly at the preclinical and formulation-development stage, although early clinical exploration has been reported in selected localized applications (NCT07088679). Nisin has been incorporated in gels and coatings for oral, peri-implant, wound-care and topical applications, extending the use of this established food-grade bacteriocin into localized healthcare contexts. A detailed list of clinical trials involving AMP candidates is provided in Supplementary Table S3.

Overall, recent AMP- based healthcare studies showed their translational potential against antibiotic-resistant infections, particularly as topical agents, antibiofilm compounds, antibiotic adjuvants and targeted microbiome-sparing antimicrobials. Nevertheless, promising in vitro or early preclinical activity does not necessarily predict clinical success. Despite antimicrobial and antibiofilm potential, the clinical translation of AMPS remains limited by interconnected safety, efficacy and scalability barriers [16]. In fact, incomplete selectivity for microbial over eukaryotic membranes may lead to cytotoxicity, hemolytic activity and, for some systemically administered AMPs such as polymyxins, clinically relevant nephrotoxicity [16]. The AMP’s in vivo efficacy may also be compromised by proteolytic degradation, loss of activity in ionic and/or protein-rich complex media, including saline or serum-containing environments, protein-binding, short half-life, pharmacokinetic/pharmacodynamic uncertainty and inefficient delivery to the infection site [16]. Moreover, clinical application is further limited by high production and purification costs, scalability issues and acquired resistance mechanisms to AMPs [16].

### 4.2. Other Healthcare Applications and Endogenous AMP Modulation

In addition to their use against antibiotic-resistant infections, AMPs are being investigated in broader healthcare contexts, including cancer, hospital hygiene, mucosal defence, inflammatory diseases and host-directed therapeutic strategies. These applications reflect the multifunctional nature of AMPs, which may combine direct antimicrobial activity with antibiofilm, immunomodulatory, wound-healing or tissue-protective effects. Moreover, AMPs are also relevant in healthcare as endogenous host-defence molecules. In this context, endogenous AMP modulation can be viewed as a host-directed antimicrobial strategy, rather than as the direct administration of an antimicrobial compound. Nutritional, microbial, hormonal and pharmacological interventions may influence AMP expression and thereby affect epithelial and mucosal antimicrobial defence, inflammation and barrier function.

Some exogenous AMPs have been evaluated for dual antimicrobial and anticancer activity. La et al. (2025) characterized brevinin-1 E8.13, isolated from the skin secretion of the Vietnamese frog *Sylvirana guentheri*, and reported antibacterial activity against *S. aureus* together with antiproliferative effects against lung, gastric, leukaemia, colorectal and liver cancer cell lines [162]. Importantly, the peptide showed comparatively low toxicity toward normal human fibroblasts [162]. Although such findings are promising, anticancer applications of AMPs remain at an early stage and require careful evaluation of selectivity, pharmacokinetics, systemic toxicity and delivery.

AMPs have also been proposed for infection prevention and hygiene applications. Asghar et al. (2025) explored purified AMPs isolated from non-resistant hospital-environment strains as alternatives to probiotic-based cleaning strategies, aiming to reduce the spread of hospital-associated resistant pathogens [163]. The authors identified *Bacillus paracheniformis* UAB33 as a producer of bacitracin B1 and reported significant reductions in microbial load when the peptide was formulated into disinfectant wipes [163]. This type of approach suggests the potential use of AMPs as components of non-living antimicrobial surface interventions, although performance under real hospital conditions still requires validation.

A growing number of clinical studies also evaluate endogenous AMPs as biomarkers of immune activation, mucosal integrity and treatment response. These studies include measurements of LL-37, hCAP18, calprotectin and other AMP-related markers in gastrointestinal, respiratory, cutaneous, urogenital and periodontal contexts. Nutritional, microbial, hormonal and pharmacological interventions, including vitamin D, omega-3 fatty acids, phenylbutyrate, probiotics, amino acid supplementation and oestrogen therapy, have been investigated for their capacity to modulate endogenous AMP expression. A detailed list of clinical trials using endogenous AMP levels as biomarkers is provided in Supplementary Table S4.

The vitamin D/LL-37 axis is particularly well represented in clinical research. Vitamin D supplementation has been associated with increased LL-37 or hCAP18 levels in serum, respiratory secretions or skin, supporting the connection between micronutrient status and AMP-mediated innate immunity [164,165]. Trials focused specifically on LL-37 and cathelicidin modulation are summarized in Supplementary Table S5. Interventions, including omega-3 fatty acids, phenylbutyrate and probiotics, have also been investigated for their potential to enhance LL-37-associated mucosal defence. Clinical contexts include tuberculosis, sepsis, chronic kidney disease, chronic obstructive pulmonary disease, peri-implantitis and inflammatory skin conditions (Supplementary Table S5). However, the clinical benefit of AMP upregulation remains context-dependent and should not be assumed solely from biomarker changes.

Gastrointestinal studies further illustrate the potential role of endogenous AMPs in mucosal defence. For example, clinical trials have evaluated faecal AMPs following probiotic intake, amino acid-fortified oral rehydration therapy or *Bifidobacterium longum* supplementation, linking these peptides to intestinal barrier function, microbial balance and host defence. One trial investigated whether isoleucine supplementation in oral rehydration solution could induce AMP production and improve outcomes in children with acute diarrhoea [166]. Although preliminary findings suggested possible benefit, larger and well-controlled trials are needed to confirm efficacy, optimal dosage, safety and mechanisms of action [166].

Cutaneous, oral and periodontal studies have also assessed AMP expression as a marker of disease activity or therapeutic response. Changes in AMP levels have been evaluated in rosacea, psoriasis, atopic dermatitis, periodontitis, peri-implantitis, hidradenitis suppurativa and other inflammatory conditions. In these settings, AMPs may reflect epithelial barrier status, microbial dysbiosis, local inflammation or response to topical and systemic treatments. However, interpretation remains complex because increased AMP expression may indicate either improved host defence or ongoing inflammatory activation, depending on the disease context.

Taken together, these studies indicate that AMPs are relevant not only as antimicrobial candidates but also as biomarkers and modulators of epithelial and mucosal immunity. Their broader healthcare value will depend on distinguishing when AMP induction is protective, neutral or pathological, and on translating biomarker associations into clinically meaningful outcomes.

### 4.3. Applications in Animal Health and Feed

The application of AMPs in animals is mainly centred on their potential use as alternatives to antibiotics in animal feed. To facilitate comparison between the AMP-based approaches discussed in this section, Table 2 summarises the animal species/model, main reported outcome and experimental stage of the studies described below.

Liu et al. (2024) evaluated the immune-enhancing effects of the heat-resistant AMP LLv, derived from human LL-37, when added to the diet of broiler chickens [167]. In that study, dietary supplementation with 100 mg/kg LLv increased serum immune indicators, including IgA, IgM, IL-4 and avian influenza virus antibody (AIV-Ab), and modulated immune-related gene expression in the jejunal mucosa [167]. These findings indicate that the immunomodulatory effect of LLv is dose-dependent and should be interpreted in relation to the dietary supplementation level used [167]. Daneshmand et al. (2020) evaluated the recombinant AMP cLFchimera, derived from camel lactoferrin, in broiler chickens challenged with necrotic enteritis [168]. The experimental AMP group received 20 mg cLFchimera/kg diet, while the antibiotic comparator group received 45 mg bacitracin methylene disalicylate/kg diet [168]. The study assessed mortality, intestinal morphology and lesions, gut microbiota balance and expression of immune and tight-junction genes [168]. The findings suggest that cLFchimera may help mitigate necrotic enteritis-associated damage and restore microbial balance in challenged birds [168]. Wang et al. (2024) explored ε-polylysine hydrochloride (ε-PLH), a natural antimicrobial peptide extracted from *Streptomyces albulus*, as a dietary supplement for laying hens [169]. Although often discussed together with antimicrobial peptides because of its peptide bonds, cationic nature and antimicrobial activity, ε-PLH is more accurately classified as a microbial poly(amino acid) antimicrobial compound rather than a conventional ribosomally synthesized AMP [170]. The study reported beneficial effects of ε-PLH supplementation on laying performance, egg quality, serum biochemical parameters, antioxidant status, intestinal morphology, gut microbiota and volatile fatty acid profiles [169]. Where reported, the active dietary level should be specified; for example, 0.05% ε-PLH supplementation was associated with improved production-related parameters and modulation of cecal microbiota in laying hens [169]. These data support the potential of ε-PLH as a feed additive, although its practical application requires optimization of dose, production stage and target outcome [169]. Importantly, ε-polylysine has an established history of food use as a preservative in several countries, including Japan, and has been the subject of FDA GRAS notices in the United States for use as an antimicrobial agent in selected food applications [170,171,172].

Jia et al. (2025) evaluated the effectiveness of the AMP R7I as an alternative to antibiotics for treating bacterial infection in geese [173]. The authors investigated an outbreak of an acute diarrheal disease in domestic geese caused by a multi-antibiotic-resistant strain of Gram-negative bacteria, *Neisseria* S1 [173].

In the in vivo model, geese were orally infected with 2 × 10^8^ CFU of *Neisseria* S1 for three consecutive days, and the treatment group received orally administered R7I at 20 mg/kg after infection, using neomycin at 20 mg/kg as the antibiotic comparator [173]. Under these conditions, R7I reduced *Neisseria* S1 infection in vitro and in vivo, supporting its potential as an oral AMP-based intervention against bacterial diarrhoeal disease in poultry [173].

Several studies have evaluated AMPs in aquaculture. Ting et al. (2018) generated transgenic *Artemia* expressing epinecidin-1 (Epi-1) and assessed whether these organisms could be used as functional feed for *Nile tilapia* fry [174]. The engineered *Artemia* enhanced resistance and survival against acute bacterial infections caused by Gram-positive and Gram-negative pathogens [174]. Wang et al. (2021) examined an AMP mixture isolated from chicken and pig intestines as a potential alternative to antibiotics in *Pengze crucian* carp [175]. The results indicated improved growth performance, digestive enzyme activity, antioxidant capacity, immune-related gene expression and resistance to *Aeromonas hydrophila* challenge [175]. These findings support the potential of AMP-based feed additives in aquaculture, although validation under commercial production conditions is still required. Another approach is to use engineered probiotics expressing AMPs. The proline-rich antimicrobial peptide PR39 is a cationic host defence peptide originally isolated from porcine leukocytes, with dual roles in antimicrobial defence and immune modulation [176]. Zhang et al. (2016) expressed a synthetic *PR39* gene in *Lactobacillus casei* 393, generating a recombinant strain capable of secreting bioactive PR39 [177]. The recombinant *L. casei* exhibited antibacterial activity against *E. coli* and *Salmonella*, but limited effects on *Staphylococcus aureus*. In vivo, BALB/c mice fed with the engineered strain showed enhanced growth, improved intestinal morphology, and increased immune cell counts, while displaying reduced mortality following enterotoxigenic *E. coli* K88^+^ challenge [177]. These findings demonstrate that PR39-expressing *L. casei* effectively combines probiotic and antimicrobial properties, supporting its potential use as an animal feed additive to promote growth and resistance to enteric infections [177]. In another study, Wen et al. (2023) constructed a recombinant *Bacillus subtilis* strain, WB800-KR32, that expresses the AMP KR32 [178]. Administration of the engineered probiotic to piglets challenged with enterotoxigenic *E. coli* alleviated intestinal oxidative injury and favourably altered the faecal microbial community [178]. Because these approaches rely on viable engineered microorganisms, their use as feed additives also raises biosafety and regulatory considerations that go beyond the activity of the expressed AMP. In addition to efficacy, future development should assess biological containment, environmental persistence, faecal shedding, horizontal gene transfer of recombinant constructs or selectable markers, and the genetic stability of the engineered strain during production, storage, gastrointestinal passage and repeated administration [177,178,179]. Strategies such as stable chromosomal integration, avoidance of antibiotic-resistance markers, minimization of mobile genetic elements and confirmation of expression stability over multiple generations may help reduce these risks [177,178]. Therefore, engineered probiotic AMP platforms should be evaluated not only for antimicrobial efficacy and host benefit, but also for strain stability, containment and regulatory safety before use in animal-production systems. Specific AMPs, including lactoferrin-derived peptides and lysozyme-based hybrid peptides, are considered potential alternatives to conventional antibiotics for reducing drug residues and pathogen resistance in animal production [180]. Sun et al. (2019) explored the hybrid AMP LfcinB-hLY, generated by fusing bovine lactoferrin and human lysozyme, as a potential related antimicrobial candidate [180]. This was primarily a recombinant production and in vitro activity study rather than a dietary-dose trial. The authors reported a LfcinB-hLY production yield of 15.7 mg/L in *Pichia pastoris*, with approximately 1.8 mg purified peptide obtained from 500 mL culture, and showed antibacterial activity against Gram-negative and Gram-positive bacteria, together with resistance to trypsin and chymotrypsin digestion under acidic conditions [180].

Zhang et al. (2018) analysed the recombinant hybrid AMP magainin II-cecropin B (MagII-CB), combining magainin II from Xenopus laevis and cecropin B from the Cecropia moth [181]. Recombinant MagII-CB was expressed in *Cordyceps militaris*, reaching 4.5 mg/g freeze-dried mycelium powder, and purified MagII-CB showed MIC values ranging from 2 to 32 µg/mL against tested Gram-positive and Gram-negative bacteria, including MICs of 2 µg/mL against *S. aureus* and 4 µg/mL against *E. coli* and *B. subtilis* [181]. In mice infected with *E. coli* ATCC 25922, MagII-CB improved intestinal barrier-associated parameters and modulated immune and inflammatory markers [181]. These results support its potential as a livestock feed additive, although further studies are needed to define optimal dosing, long-term safety and efficacy under production conditions [181].

From a feed-application perspective, these studies suggest that AMPs may influence animal health not only through direct antimicrobial activity, but also by modulating gut microbiota composition, intestinal barrier function, oxidative stress and immune responses [182,183]. However, their practical use as feed additives requires careful consideration of formulation and production constraints. AMPs must remain active during feed processing and storage, including exposure to heat, pressure, moisture and interactions with feed components [182,183]. They must also resist, or be protected from, degradation by gastrointestinal proteases, while maintaining activity at doses that are effective, economically feasible and safe for the target species [182,183]. In addition, residue formation, potential effects on non-target microbiota, toxicity, immunogenicity and the risk of resistance development should be evaluated before large-scale implementation [184]. Therefore, future studies should define optimal dosage, delivery format, processing stability, digestive stability and safety profiles under realistic animal-production conditions.

## 5. Agricultural Applications of Natural Antimicrobials

Natural antimicrobials are being explored as lower-residue and more targeted alternatives or complements to conventional pesticides in crop protection. Within the scope of this review, particular attention is given to AMPs, bacteriocins, mycocins and related peptide-based antimicrobial compounds with activity against phytopathogenic bacteria and fungi. However, agricultural translation must be interpreted according to the application context. Evidence obtained in vitro or under controlled conditions cannot be assumed to predict open-field performance, where UV exposure, rainfall, temperature variation, plant-surface microbiota, formulation stability and persistence on leaves or fruit surfaces strongly influence efficacy. Therefore, this section distinguishes controlled-environment or greenhouse applications, open-field crop-protection examples and postharvest uses.

### 5.1. Controlled Environment and Greenhouse Applications

Controlled environment and greenhouse studies are important intermediate steps between in vitro antimicrobial screening and field deployment. In these systems, temperature, humidity, inoculum pressure and treatment timing can be more tightly controlled, allowing a clearer assessment of antimicrobial activity on plants. Bacteriocin-producing rhizobacteria and other antimicrobial-producing bacteria have shown activity against plant-pathogenic bacteria such as *Erwinia*, *Xanthomonas* and *Pseudomonas* spp., including effects on growth inhibition, biofilm formation and disease suppression in planta [185,186].

Specific examples support the agricultural relevance of bacteriocin-mediated disease suppression. In rice, non-pathogenic bacteriocinogenic strains of *Xanthomonas oryzae* pv. *oryzae* were tested against bacterial blight caused by virulent *X. oryzae* pv. *oryzae*, reducing disease incidence in greenhouse and screenhouse experiments [187]. In olive, a bacteriocin produced by *Pseudomonas syringae* pv. *ciccaronei* NCPPB2355 inhibited *Pseudomonas savastanoi* pv. *savastanoi* (formerly described as *P. syringae* subsp. *savastanoi*) the causal agent of olive knot disease, and reduced knot formation and epiphytic survival of the pathogen on treated olive plants [188]. Earlier studies also reported that avirulent bacteriocin-producing strains of *Ralstonia solanacearum* (formerly *Pseudomonas solanacearum*) could reduce bacterial wilt development in solanaceous crops such as tobacco and tomato [189].

These examples show that bacteriocin-based plant-disease suppression has been demonstrated in specific pathosystems. However, the evidence often involves producer strains, avirulent strains or crude bacteriocin preparations rather than purified, formulation-ready bacteriocins. This distinction is important because plant protection may result from multiple mechanisms, including antibiosis, competition, biofilm interference, induced systemic resistance and plant-growth promotion [185,186,190].

Mycocins also provide relevant examples under controlled plant-disease conditions. The killer toxin produced by *Pichia membranifaciens* CYC 1106 inhibited *Botrytis cinerea*, the causal agent of grey mould, and reduced disease development in treated *Vitis vinifera* plants under experimental conditions [191]. This example supports the potential of yeast killer toxins as antifungal biocontrol tools, but it should still be interpreted as controlled-condition evidence rather than broad field validation.

Microbial lipopeptides provide another important class of peptide-like antimicrobials for greenhouse applications. Cell-free supernatants from *Bacillus subtilis* ET-1 containing iturin A were tested against powdery mildew caused by *Podosphaera xanthii* on melon plants (*Cucumis melo*) under greenhouse conditions [192]. In that study, treatments containing 400, 40 and 4 mg/L iturin A produced dose-dependent disease-control effects, with complete disease control reported at the highest concentration tested [192]. This type of evidence is particularly useful because it links a defined lipopeptide-rich preparation with a plant-pathogen system and a measurable in planta disease outcome.

Plant-derived AMPs have also been investigated as topical biofungicide candidates. Cyclotides, which are small cysteine-rich cyclic peptides, have attracted attention because of their structural stability and pesticidal or antifungal activities [193]. Similarly, Aq-AMP from *Amaranthus quitensis* showed antifungal activity against phytopathogens such as *Penicillium*, *Fusarium* and *Aspergillus* spp., together with thermal stability and low cytotoxicity in the reported models [194]. These examples indicate potential for agricultural use, but most evidence remains closer to laboratory or controlled-condition validation than to full field deployment.

Synthetic and engineered antimicrobial peptides have also been screened against bacterial plant pathogens such as *Xanthomonas*, *Erwinia* and *Pseudomonas* spp. [195]. These peptides may inhibit bacterial growth, interfere with biofilm formation and, in some cases, act as plant-defence elicitors [195]. However, because these molecules are synthetic or engineered, they should be considered translational analogues inspired by natural peptide scaffolds rather than natural antimicrobials in the strict sense. Their agricultural use also requires field-specific validation of phytotoxicity, persistence, environmental fate, production cost and regulatory classification [195].

### 5.2. Open-Field Crop-Protection Applications

Open-field application is a more demanding stage of validation. Peptide-based antimicrobials applied to crops are exposed to sunlight, rainfall, wash-off, oxidation, proteolysis, variable humidity, microbial degradation and uneven distribution on plant surfaces. For this reason, open-field evidence remains more limited than in vitro and greenhouse evidence, especially for purified AMPs, bacteriocins and mycocins.

A useful field-relevant example is the work with *B. subtilis* ET-1 lipopeptide preparations against melon powdery mildew. After greenhouse testing, both cell-free supernatant and crude lipopeptide extract containing approximately 100 ppm iturin A were evaluated under open-field conditions on melon plants artificially inoculated with *P. xanthii* [192]. Both treatments showed strong disease control, comparable to that obtained with a conventional chemical fungicide in that study [192]. This example is important because it moves beyond in vitro inhibition and greenhouse assays. Nevertheless, it also illustrates a broader limitation of the field: the active treatment was a lipopeptide-rich preparation derived from a microbial producer strain, not a single purified peptide product.

Commercially used *Trichoderma*-based biofungicides illustrate the distinction between producer-organism biocontrol and the direct application of purified antimicrobial peptides. *Trichoderma* spp. can produce peptaibols and other antimicrobial metabolites with activity against phytopathogenic fungi [196]. However, the efficacy of *Trichoderma*-based products in agricultural systems is multifactorial and should not be attributed only to peptaibols. Their biocontrol activity may involve competition for nutrients and space, rhizosphere or phyllosphere colonisation, mycoparasitism, secretion of lytic enzymes, antibiosis, production of secondary metabolites and induction of local or systemic plant defence responses [196,197]. Therefore, these products support the relevance of peptide-producing microorganisms in agriculture, but they should not be presented as direct field applications of purified peptaibols alone.

### 5.3. Postharvest Applications

Postharvest applications represent a distinct agricultural context. In this case, antimicrobial treatments are applied after harvest to fruit, vegetables or storage environments to reduce spoilage during transport, storage or marketing. These systems are often more controlled than open-field applications, because temperature, humidity, treatment concentration and exposure time can be better managed. They are therefore particularly relevant for peptide-based antifungal agents targeting wound-invading or storage-associated pathogens.

Yeast killer toxins and mycocins have been explored in this setting. *Pichia membranifaciens* and its purified killer toxin showed inhibitory effects against *B. cinerea* and reduced disease development in postharvest apple wound experiments [198]. This provides a clearer postharvest example than many general claims about mycocin activity, because it links a defined yeast killer system with a relevant fruit pathogen and an in vivo fruit model. However, broader use will require formulation, stability and safety validation under commercial storage conditions.

Lipopeptides from *Bacillus* spp. also show postharvest potential. Lipopeptides produced by *B. subtilis* Y17B, including surfactin, iturin and fengycin families, inhibited *Alternaria alternata* and reduced postharvest *Alternaria* fruit rot of cherry in fruit assays [199]. Microscopy-based analyses in that study indicated fungal structural damage, supporting a direct antifungal effect of the lipopeptide preparation [199]. This example is useful because it addresses a real postharvest disease system rather than only laboratory growth inhibition.

Aq-AMP and other plant-derived antifungal peptides may also be relevant for postharvest disease control, particularly when applied as topical treatments against fungal spoilage organisms [194]. However, as with other peptide-based antimicrobials, evidence should be interpreted according to the level of validation: in vitro inhibition, treated fruit assays, pilot storage studies and commercial postharvest application are not equivalent stages of development.

Overall, agricultural applications of AMPs, bacteriocins, mycocins and related peptide-based antimicrobials remain promising but unevenly developed. The strongest evidence is often found in in vitro assays, controlled plant experiments, greenhouse trials and postharvest models, whereas open-field validation remains more limited and frequently involves antimicrobial-producing microorganisms, cell-free supernatants or crude peptide/lipopeptide extracts rather than purified antimicrobial peptides. Future studies should therefore clearly distinguish purified peptide activity, producer-strain biocontrol, greenhouse efficacy, field performance and postharvest protection. Key translational challenges include formulation stability, persistence on plant surfaces, resistance to UV exposure and rainfall, phytotoxicity, effects on beneficial microbiota and pollinators, production cost, environmental fate and regulatory approval as plant-protection or postharvest biocontrol products.

### 5.4. Environmental Fate, Ecotoxicity and Non-Target Effects

The environmental advantages of peptide-based natural antimicrobials should be interpreted cautiously. Their biodegradability and targeted activity may reduce long-term chemical residues compared with some conventional pesticides, but these properties do not automatically guarantee environmental safety [200,201]. After agricultural application, AMPs, bacteriocins, mycocins and lipopeptide-rich preparations may be exposed to UV radiation, rainfall, adsorption to soil particles, enzymatic degradation, microbial metabolism and wash-off into surrounding environments. Their environmental fate will depend on peptide structure, formulation, dose, application frequency, crop surface, soil type, climate and persistence of the delivery system [200,202].

Formulation is particularly important. Encapsulation, coatings, wetting agents, stickers or slow-release systems may improve field stability and rainfastness, but they may also prolong environmental persistence and increase exposure of non-target organisms [201,202]. Therefore, formulation strategies should be evaluated not only for efficacy against plant pathogens but also for degradation profile, mobility, accumulation potential and release behaviour under realistic field conditions [200,202].

Ecotoxicological assessment is also necessary. Although many peptide-based antimicrobials are expected to degrade more readily than persistent synthetic pesticides, they may still affect non-target microorganisms or beneficial organisms depending on spectrum of activity, environmental concentration and exposure route [200,201]. Particular attention should be given to soil and phyllosphere microbiota, plant-growth-promoting bacteria, mycorrhizal fungi, pollinators, aquatic organisms and natural enemies used in integrated pest management [200,201]. For microbial producer strains or crude extracts, risk assessment should also consider strain persistence, possible genetic exchange, production of secondary metabolites and effects on native microbial communities [201,202,203,204].

Therefore, environmental benefits should be considered potential advantages rather than assumed outcomes. Broader agricultural implementation will require field-level studies addressing environmental fate, ecotoxicity, persistence of formulations, impact on non-target microbiota, effects on beneficial insects and compatibility with integrated pest management [200,201,202,203]. These data are essential to define safe application rates, re-entry intervals, preharvest intervals, formulation design and regulatory classification as plant-protection or postharvest biocontrol products [202,203].

## 6. Production and Bioengineering Strategies for Natural Antimicrobials

The natural production of AMPs, bacteriocins and mycocins in living organisms is often limited by low yields, tight regulation, susceptibility to proteolytic degradation, toxicity to host cells and high manufacturing costs [205,206]. Several strategies have been developed to enhance antimicrobial peptide production in both native and heterologous systems [205,206]. The following section summarizes strategies to improve yield, stability, safety and delivery of natural antimicrobial peptides and related compounds, including endogenous AMP upregulation and formulation/delivery approaches [207,208].

### 6.1. Strategies to Enhance Natural Production

At the organismal level, nutritional, microbial, hormonal and environmental stimuli can upregulate innate AMP expression [207,209,210,211]. These approaches are mainly relevant to host-directed or in vivo enhancement of endogenous antimicrobial defence, rather than to the industrial manufacture of purified antimicrobial compounds. They are included here to illustrate how natural AMP production can be stimulated within living systems, complementing downstream biotechnological strategies such as recombinant expression, synthetic biology and optimized fermentation. For example, Vitamin D_3_ enhances transcription of *CAMP* through the vitamin D receptor pathway [207], whereas short-chain fatty acids, including butyrate and propionate, can increase defensin and cathelicidin expression in epithelial cells through histone deacetylase inhibition [209,210]. Similarly, probiotic or microbial components such as lipopolysaccharides, peptidoglycans and flagellins activate pattern-recognition receptors (PRRs), including Toll-like receptors and NOD-like receptors, to trigger AMP synthesis in mucosal tissues [211].

In parallel, advances in metabolic and synthetic biology enable the improvement of AMP yield in natural producers by manipulating regulatory elements, promoters, signal peptides and precursor-processing enzymes [205,206]. For example, Zhao et al. (2015) engineered a recombinant fusion protein combining parasin I (PI), a catfish-derived AMP, with human lysozyme (hLY) to enable safe and efficient production in Pichia pastoris [212]. The authors reported improved secretion of a protein that retained activity after enzymatic cleavage and showed synergistic antibacterial effects, suggesting potential application as a feed additive [212]. Dong et al. (2023) established a recombinant Pichia pastoris expression system to produce Turgencin A, a peptide that is difficult to isolate naturally and less active when obtained by chemical synthesis [213]. Recombinant Turgencin A showed broad-spectrum antimicrobial activity against Gram-negative and Gram-positive bacteria and potential application as a food preservative in pork [213].

In plant systems, treatment with jasmonic acid or salicylic acid analogues can elicit AMP accumulation as part of systemic acquired resistance [214,215]. Together, these physiological and molecular strategies aim to exploit the organism’s intrinsic defence pathways to obtain higher peptide yields in a sustainable and biologically relevant manner, reducing dependence on costly chemical synthesis or recombinant overexpression systems [207,209,210,211,212,213,214,215].

### 6.2. Production of Synthetic AMP Analogues

Peptoids and other synthetic AMP analogues are being developed to improve antimicrobial efficacy, stability and selectivity by targeting bacterial membranes and intracellular components [216,217]. Modifications may include deletions, substitutions, changes in physicochemical properties or secondary structure, C-terminal amidation, methylation, dimerization, hybridization or incorporation of non-natural building blocks [217]. Nielsen et al. (2022) and Wardell et al. (2025) evaluated cationic amphipathic peptoids TM1–TM20, designed to self-assemble into defined nanostructures [218,219]. Lipo-peptoid TM18 showed antimicrobial, antibiofilm and anti-abscess activity against multidrug-resistant *P. aeruginosa* and MRSA [218]. Rahman et al. (2025) synthesized 15 analogues of the lipopeptide Humimycin A and found that the β-hydroxymyristoyl lipid chain and C-terminal carboxylic acid were critical for activity against *S. aureus* [220].

Zhang et al. (2025) used a template-based de novo design strategy to build α-helical antimicrobial peptides with repetitive subunits (XXFY)n and a β-sheet comparator (KFKY)n, systematically mapping relationships between structure and activity against susceptible and resistant bacteria [221]. Peptide 27 (OOFI4, where O is ornithine, F is phenylalanine and I is isoleucine) showed bactericidal, membrane-disrupting and immunomodulatory activity and was effective in murine models of multidrug-resistant *P. aeruginosa* and MRSA-induced skin infections [221]. Li et al. (2025) analysed a library of β-hairpin AMPs with identical β-turn sequences and varying length and arrangement of alternating hydrophobic and hydrophilic residues [222]. They identified the D-amino acid sequence D-G(RF)3 as having high efficacy, low toxicity, stability against digestive enzymes and mouse plasma, antibacterial and antibiofilm activity, in vivo efficacy in a mouse pulmonary infection model and synergy with conventional antibiotics [222]. Kim et al. (2025) evaluated 13-amino acid AMPs for *acne vulgaris* by varying the number and position of tryptophan residues [223]. Two peptides showed activity against susceptible and resistant *Cutibacterium acnes* strains, reduced *C. acnes*-induced ear swelling in a mouse model and exhibited low dermal-cell cytotoxicity, low induction of pro-inflammatory cytokines and stability for up to 12 h after protease exposure [223].

### 6.3. Nanovectorization

Nanotechnology strategies are also being used to improve the antimicrobial performance stability, delivery and safety profile of natural peptides [224,225,226,227,228,229]. Liu et al. (2025) combined black phosphorus nanosheets (BPs) with the AMP melittin to generate BPs/Mel nanocomposites and evaluated their antimicrobial activity against *Escherichia coli* and *Staphylococcus aureus* [224]. The most favourable formulation, BPs/Mel-7, contained middle-sized BPs and showed IC_50_ values of 19.1 ± 2.8 µg/mL against *E. coli* and 12.6 ± 1.3 µg/mL against *S. aureus*, whereas BPs alone showed no detectable antibacterial activity at concentrations up to 200 µg/mL [224]. In live/dead staining assays, BPs/Mel-7 at 50 µg/mL killed more than 80% of bacterial cells, and almost complete bacterial killing was observed when the concentration was increased to 100 µg/mL [224]. In an *S. aureus*-infected mouse wound model, wounds were treated with 200 µL of BPs/Mel-7 at 50 µg/mL, resulting in approximately 90% wound closure after 7 days, reduced bacterial burden and lower IL-6 and TNF-α levels compared with controls. Xiao et al. (2025) functionalized zeolitic imidazolate framework-8 (ZIF-8)-coated MnO_2_ nanoparticles with an antibacterial peptide to generate AP-MnO_2_@ZIF-8 [225]. This nanocomposite showed MIC values of 20 µg/mL against multidrug-resistant *E. coli* and 39 µg/mL against *S. aureus* [225]. In colony-forming unit assays, 150 µg/mL AP-MnO_2_@ZIF-8 reduced bacterial survival below 5%, indicating strong growth inhibition under the tested conditions [225]. The nanocomposite also suppressed bacterial activity in an *S. aureus*-infected mouse wound model, supporting its potential as an AMP-based wound-healing platform [225]. Li et al. (2025) proposed a metal-organic framework (UiO-66)-based nanoplatform functionalized with the AMP UBI29-41 and loaded with indocyanine green (ICG) to enhance near-infrared-triggered antimicrobial photodynamic therapy against periodontal biofilms [226]. The ICG@UiO-66-UBI nanocomposite was evaluated at concentrations up to 50 µg/mL for cytocompatibility, with cell viability remaining above 90% after 24 h at concentrations ≤ 40 µg/mL; 30 µg/mL was selected as a relatively biosafe concentration for subsequent experiments [226]. Under 808 nm near-infrared irradiation, ICG@UiO-66-UBI reduced colony-forming units in single-species periodontal biofilms by approximately two orders of magnitude and disrupted quorum-sensing-associated pathways, including the LuxS/AI-2 system and related virulence genes [226]. These effects were linked to UBI29-41-mediated targeting of bacterial surface components and ICG-generated ROS under near-infrared irradiation [226].

Kłodzińska et al. (2025) used hyaluronic acid nanogels modified with octenyl succinic anhydride to improve the pharmacokinetics and safety profile of human LL-37 [227]. After radiolabelling the AMP with gallium-67 and the polymer with indium-111, the nanoformulation was tracked in mice after intratracheal administration using single-photon emission computed tomography (SPECT) [227]. The nanoformulation improved AMP retention in the lungs and reduced accumulation in excretory organs, potentially lowering kidney and liver toxicity [227]. Yang et al. (2025) engineered an LPS-targeting, non-lytic M13 phage conjugated with polymyxin B (PMB-M13aLPS) [228]. This platform delivered the peptide specifically to Gram-negative bacteria, reduced MICs by two orders of magnitude and cured MDR *P. aeruginosa* infections in mice with improved potency and tolerability compared with PMB alone [228].

Another strategy involves structurally nanoengineered antimicrobial peptide polymers (SNAPPs). First introduced by Lam et al. (2016), SNAPPs consist of a star-like architecture composed of a nanoengineered core with radiating peptide arms [229]. Their efficacy is influenced by the number, length and amino acid sequence of these arms [230]. Jayawardena et al. (2025) simulated the molecular dynamics of SNAPPs interacting with bacterial membranes [231,232]. One study compared alternating, random and diblock arrangements of lysine (K), a hydrophilic positively charged amino acid, and valine (V), a hydrophobic amino acid [232]. SNAPPs with alternating or random blocks adopted an “octopus-like” configuration after membrane contact, leading to submersion, water infiltration and pore formation [232]. In contrast, diblock SNAPPs maintained a “pufferfish-like” morphology due to electrostatic repulsion, resulting in weak adherence and limited water penetration [232]. A second study examined lipidation effects on α-helical stability and membrane disruption, identifying C12-SNAPP as the most disruptive configuration and C18-SNAPP as less effective because of hydrophobic hindrance.

As a complementary approach, nanoparticle carriers have also been explored for CRISPR/Cas9 delivery to disrupt biofilm-associated resistance or quorum-sensing genes [233,234,235,236]. Although CRISPR/Cas9 is not a natural antimicrobial compound, these systems are briefly mentioned here because they illustrate how nano-enabled platforms may support combination strategies against drug-resistant biofilms, including co-delivery with antimicrobial agents [233,234,235,236].

## 7. Challenges and Future Perspectives

Despite the growing interest in AMPs, bacteriocins and mycocins as natural antimicrobial agents, their widespread application in food safety, agriculture and healthcare remains limited by biological, technological and regulatory challenges. These compounds offer important advantages, including structural diversity, biodegradability, target specificity and multiple mechanisms of action [31,36,185]. However, antimicrobial activity demonstrated under controlled laboratory conditions is not sufficient to ensure successful translation into industrial or clinical applications. Their effectiveness must be confirmed in complex matrices, under realistic processing and storage conditions, and within regulatory frameworks that differ between food, cosmetic, agricultural, veterinary and pharmaceutical sectors [14,16,121,122,123,237,238].

One major challenge is the possible emergence of microbial resistance or evasion mechanisms. Although AMPs, bacteriocins and mycocins are often considered less prone to resistance development than conventional antibiotics, this risk should not be underestimated [12,238,239,240,241]. Microorganisms may reduce susceptibility by modifying membrane charge, altering cell wall or outer membrane composition, changing surface receptors, producing proteases, activating efflux systems or forming biofilms that restrict peptide penetration [239,240,241,242]. For narrow spectrum bacteriocins, receptor modification or loss may be sufficient to reduce efficacy [12,242,243]. Future studies should therefore assess not only immediate antimicrobial activity but also resistance development after serial exposure, possible cross-resistance with conventional antibiotics and effects on non-target microbial communities [133,239,240,241,242].

Biofilms further complicate antimicrobial application. Many spoilage and pathogenic microorganisms persist in biofilms on food-processing surfaces, medical devices, wounds, plant tissues and animal production environments. Biofilm matrices can limit peptide diffusion, reduce access to target cells and promote phenotypic tolerance [243]. Although several AMPs and bacteriocins show antibiofilm activity, their efficacy depends strongly on the target species, biofilm maturity, matrix composition and delivery system [133,243,244,245,246,247]. This supports the use of realistic biofilm models rather than relying only on planktonic assays. Combination strategies, including AMPs with conventional antibiotics, mild processing technologies, enzymes, organic acids, nanocarriers or quorum-sensing inhibitors, may be more effective than single-agent approaches [35,133,226,246].

Stability is another critical limitation. Many AMPs, bacteriocins and mycocins are sensitive to proteolytic degradation, oxidation, pH changes, temperature variation, ionic strength and interactions with proteins, lipids or polysaccharides [15,76,246]. In food matrices, antimicrobial peptides may bind to fat or protein fractions, reducing their availability and activity [14,83,127]. In cosmetic formulations, they may interact with surfactants, emulsifiers, preservatives or charged polymers [124,125,126]. In healthcare, they may be degraded by host proteases, rapidly cleared or inactivated by physiological salts and serum components [16,31,125]. In agriculture, UV radiation, humidity, temperature changes and plant surface chemistry may further compromise stability [199,208]. Therefore, activity observed in buffer or culture medium cannot be directly extrapolated to final products or biological systems.

Formulation technologies will be central to overcoming these limitations. Encapsulation in liposomes, polymeric nanoparticles, nanogels, hydrogels, edible films, electrospun fibres or biopolymer-based coatings can protect peptides from degradation, improve solubility, reduce undesirable matrix interactions and enable controlled release [83,84,85,208,241,245]. These strategies are particularly relevant for bacteriocins such as nisin, whose activity can be reduced in complex food or topical systems [83,84,124,126,127]. Encapsulation may improve antimicrobial persistence during storage, reduce the required dose and increase local activity in food, cosmetic, healthcare and agricultural applications [83,84,85,124,126,208,227,245]. However, these systems also add complexity, cost and regulatory considerations, and their effects on release kinetics, bioavailability and safety must be evaluated case by case [125,245].

Large-scale production and quality control remain major bottlenecks. Natural producers often synthesize AMPs, bacteriocins and mycocins at low concentrations, with yields affected by strain, medium composition, fermentation conditions and downstream processing [205,206]. Purification can be expensive, especially when high purity is required for medical or pharmaceutical applications. Peptide recovery may also be affected by proteolysis, adsorption to biomass, co-production of other metabolites or instability during processing [205,206]. Recombinant expression, synthetic biology, promoter engineering, optimized signal peptides, protease-deficient hosts and low-cost substrates may improve yields and reduce costs [205,206,212,213]. Nevertheless, industrial production must ensure batch-to-batch consistency, reproducible biological activity and absence of toxic contaminants or undesirable by-products. This requires robust chemical and functional characterization, including molecular mass determination, purity assessment, sequence confirmation where applicable, antimicrobial potency, storage stability and compatibility with the intended matrix [15,64,76]. For peptide fractions or crude extracts, defining the active component, or at least a reproducible chemical and functional profile, becomes essential [61,64]. Matrix-related stability is another major technological barrier. The activity of AMPs, bacteriocins and mycocins in real products depends not only on antimicrobial potency measured in buffer or culture medium, but also on pH, ionic strength, water activity, fat content, protein content, enzymes, salts, surfactants, polyphenols, processing temperature, storage conditions and product microstructure [14,76,246]. In food systems, peptides may bind to caseins, whey proteins, meat proteins or polysaccharides, partition into fat phases, aggregate, precipitate, adsorb to packaging materials or become unevenly distributed within the matrix. In cosmetic and pharmaceutical formulations, surfactants, oils, polymers, preservatives, humectants and packaging materials may similarly alter peptide solubility, stability and availability. In plant-protection systems, adsorption to cuticular waxes, soil particles or plant residues, together with UV exposure, rainfall and microbial degradation, may reduce persistence or alter release. Consequently, matrix-specific challenge tests, formulation optimisation and measurement of active peptide availability at the target site are essential for technological translation.

Resistance and tolerance should also be considered in a compound- and context-specific manner. For bacteriocins, resistance may arise through modification, loss or reduced expression of cell-surface receptors. For example, nisin and related lantibiotics interact with lipid II, whereas several class IIa pediocin-like bacteriocins require the mannose phosphotransferase system as a receptor; changes in receptor availability or expression can therefore reduce susceptibility [74,247]. In *L. monocytogenes*, nisin resistance has also been associated with altered cell-envelope regulation, including mechanisms involving increased *pbp2229* expression mediated by the two-component system Hpk1021 [80]. More broadly, AMP tolerance mechanisms may include changes in cell-surface charge, modification of membrane lipid composition, thickening or remodelling of the cell wall, activation of stress-response pathways, efflux, proteolytic degradation, extracellular trapping or sequestration and biofilm-associated protection [239,240,242,243,248,249]. These examples show that peptide-based antimicrobials may have lower or different resistance risks than conventional antibiotics, but resistance or tolerance should not be dismissed.

Biofilm-related efficacy also requires realistic testing models. Static microtiter-plate assays are useful for initial screening, but they do not reproduce the complexity of biofilms in food-processing facilities, medical-device surfaces or plant environments. In food-processing contexts, models should include relevant materials such as stainless steel, plastic, rubber, conveyor-belt surfaces or packaging materials, mixed-species communities, organic residues, low-temperature conditions and repeated cleaning/disinfection cycles [250]. In healthcare contexts, antibiofilm testing should consider catheter, implant, wound-dressing or medical-device materials, conditioning films, flow conditions and host-relevant fluids [251]. In agriculture, plant-surface models should reflect phyllosphere, rhizosphere, leaf, fruit or root conditions, including surface topography, humidity, UV exposure, wash-off and interactions with resident microbiota [252,253]. Therefore, antibiofilm activity should be validated in application-relevant models before being used to support food, medical or agricultural translation.

Safety assessment is non-negotiable. Although many natural antimicrobials are biodegradable and some producing organisms have GRAS or QPS status, this does not automatically guarantee the safety of every peptide, extract, formulation or route of exposure [15,69,112,113]. Cytotoxicity, haemolytic activity, allergenicity, immunogenicity, pro-inflammatory effects, effects on beneficial microbiota and environmental impact must be carefully assessed [15,16,124,125]. For cosmetic and topical applications, skin irritation, sensitization and cutaneous microbiome effects must be considered [124,125]. For food applications, digestibility, gastrointestinal stability, exposure levels and interactions with food components are relevant [14,15]. For agricultural applications, effects on soil microbiota, beneficial insects, plants and aquatic systems should be addressed [200,201,202,203]. For fungal- and yeast-derived antimicrobials, the producing organism and possible co-production of undesirable secondary metabolites also require attention [65,203,204].

Regulatory approval remains one of the most complex barriers. The same molecule may be regulated differently depending on whether it is used as a food preservative, food contact material component, animal feed additive, cosmetic ingredient, plant protection product, veterinary product or pharmaceutical agent [16,121,123,237,238]. Food applications require evidence of technological need, consumer safety, toxicological profile, exposure levels and stability in the final product [238]. Agricultural applications require data on environmental fate, ecotoxicity and non-target effects [200,201,202,203,237]. Pharmaceutical applications require pharmacokinetic, pharmacodynamic, toxicity and efficacy data, often followed by clinical validation [16,35,125]. Cosmetic applications require safety assessment under expected conditions of use and compatibility with the final formulation [121,122,124,125]. Regulatory planning should therefore begin early in development.

Finally, a persistent gap remains between laboratory efficacy and real-world performance. Many studies use purified peptides, standard strains and simplified assays, while commercial applications involve complex microbial communities, organic matter, processing stresses, environmental variability and economic constraints [14,78,246]. Future studies should include food challenge tests, mixed-species biofilms, contaminated surfaces, plant-pathogen systems, skin and intestinal cell models, animal models and pilot-scale trials [15,124,133,195,250,251,252,253]. Progress will likely depend on integrated strategies combining peptide engineering, encapsulation, nanodelivery, synthetic biology, combination treatments, omics-based screening, molecular modelling and machine learning [35,133,205,206,208,216,217,224,225,226,227,228,229,231,232,254]. However, these technologies must be accompanied by toxicological evaluation, cost analysis and regulatory feasibility assessment.

Overall, AMPs, bacteriocins and mycocins should not be presented as simple replacements for conventional antibiotics, preservatives or pesticides. Their most realistic value lies in their use as targeted, biodegradable and adaptable antimicrobial tools within broader preservation, therapeutic and biocontrol strategies [12,31,36,185]. Future development should therefore be assessed not only by antimicrobial potency, but also by the ability of these compounds to overcome translational barriers, including scalable production, purification, formulation stability, safety, regulatory compliance, matrix-specific efficacy, environmental fate and cost-effective implementation.

## 8. Conclusions

Natural antimicrobials, including AMPs, bacteriocins and mycocins, represent a diverse and biologically relevant group of compounds with potential applications in food safety, agriculture, cosmetics, animal health and human healthcare. Their natural origin, structural diversity and multiple mechanisms of action make them attractive potential alternatives or complements to conventional antibiotics, synthetic preservatives and chemical pesticides. By disrupting microbial membranes, inhibiting cell wall synthesis, interfering with intracellular targets, modulating immune responses or compromising fungal cell wall integrity, these compounds offer several routes for controlling pathogenic and spoilage microorganisms.

However, their translational maturity differs considerably between classes and application areas. Bacteriocins currently appear closest to commercial implementation, particularly in food preservation, where nisin remains the most mature example because of its established industrial use and regulatory acceptance. In the short to medium term, the most realistic applications of AMPs, bacteriocins and mycocins are likely to include food biopreservation, active packaging, topical or localized antimicrobial use, cosmetic preservation and postharvest protection. Yeast-derived AMPs and mycocins also have relevant potential in fermented foods, cosmetics, postharvest systems and localized antifungal applications, but remain less developed because many active compounds still require purification, molecular identification, standardized activity measurement and validation in real matrices. Synthetic, engineered and nanoformulated AMPs may offer longer-term potential in healthcare and antibiofilm applications, but their translation is limited by proteolytic degradation, toxicity, pharmacokinetics, production costs and regulatory complexity.

In food systems, bacteriocins and yeast-derived antimicrobial peptides may improve microbial safety, extend shelf life and support clean-label preservation strategies. Nisin remains the most established example, but other bacteriocins, mycocins and antifungal peptides are increasingly being investigated for use in dairy products, meat, beverages, bakery products, fruit and vegetable preservation and active packaging. In cosmetics and topical products, peptide-based antimicrobials may act as formulation preservatives, preservative boosters or multifunctional ingredients with antimicrobial, anti-inflammatory, skin-protective or microbiome-modulating properties, provided that claims remain aligned with the appropriate regulatory category. In agriculture, AMPs, bacteriocins and mycocins may contribute to biopesticide, biofungicide and postharvest protection strategies, although field validation, formulation stability, ecotoxicity and effects on non-target microbiota remain critical requirements. In healthcare, these molecules are being explored as anti-infective agents, antibiotic adjuvants, antibiofilm compounds, immunomodulators and microbiome-sparing alternatives for localized therapy.

Despite this broad potential, translation into commercial and clinical products remains uneven. The main obstacles include limited stability, proteolytic degradation, possible toxicity, narrow or context-dependent activity, production costs, formulation difficulties, regulatory complexity and the need for validation in realistic systems. These challenges are particularly relevant when moving from purified compounds and laboratory assays to complex food matrices, cosmetic formulations, biological tissues, environmental settings or industrial-scale processes.

Future development should therefore focus on integrated solutions combining peptide engineering, recombinant production, encapsulation, nanodelivery, standardized safety assessment and well-designed efficacy studies. Greater attention should also be given to resistance development, microbiome impact, environmental fate and regulatory classification from the earliest stages of product development.

In conclusion, AMPs, bacteriocins and mycocins should be viewed not as simple replacements for conventional antimicrobial strategies, but as targeted, biodegradable and adaptable tools within broader preservation, therapeutic and biocontrol approaches. Their future impact will depend on the capacity to move beyond proof-of-concept antimicrobial activity and deliver stable, safe, scalable and regulatory-compliant solutions for real-world applications.

## Figures and Tables

**Table 1:** Representative clinical and translational applications of antimicrobial peptides in healthcare.

AMP/Compound	Type/Source	Main Application/Target Organism/Administration	Administration Route/Development Stage	Translational Relevance/Main Limitations
Colistin/polymyxin B	Cyclic lipopeptide AMPs from *Paenibacillus* spp.	Severe multidrug-resistant Gram-negative infections/targets MDR Gram-negative bacteria, including *Pseudomonas aeruginosa*, *Acinetobacter baumannii* and *Klebsiella pneumoniae*	Intravenous, nebulized, oral or topical, depending on formulation/Approved use; multiple clinical trials	Last-resort therapy/limited by nephrotoxicity, neurotoxicity and resistance concerns
C16G2	Specifically targeted antimicrobial peptide—STAMP	Dental caries/selective targeting of *Streptococcus mutans*	Topical/oral dental formulations/Phase II clinical trials	Microbiome-sparing targeted antimicrobial strategy/long-term efficacy and implementation require further validation
TAPS-18	Synthetic cathelicidin-based AMP	Periodontitis, as adjunct to non-surgical periodontal therapy/targets periodontal/oral infection-associated bacteria	Topical gel/Early-phase clinical trial	Topical/local AMP therapy for oral infections/broader efficacy requires validation in larger studies
PL-5/peceleganan	Hybrid cecropin–melittin-derived AMP	Mild infected diabetic foot ulcers/targets wound-associated bacteria in infected diabetic foot ulcers	Topical spray/Phase II clinical trial	Topical wound antimicrobial with antibiofilm potential/clinical translation depends on safety and efficacy confirmation
PL-18	Synthetic AMP formulation	Bacterial vaginosis and mixed vaginal infections	Vaginal suppository/Phase I clinical trial	Mucosal AMP formulation with potential microbiota-preserving activity/limited by early-stage safety, tolerability and PK data
Nisin-based formulations	LAB-derived bacteriocin	Oral, peri-implant, wound-care or topical antimicrobial applications	Local gels or coatings/Preclinical and formulation-stage evaluation.	Established food-grade antimicrobial being repurposed for localized healthcare uses/formulation stability, delivery and clinical efficacy require validation
LL-37/hCAP18	Human endogenous cathelicidin	Biomarker and modifiable host-defence AMP/associated with mucosal defence and inflammation	Endogenous AMP measured in biological samples or modulated by nutritional/pharmacological interventions/Clinical biomarker/intervention studies	Links innate immunity, vitamin D axis, mucosal defence and inflammatory status/clinical benefit of modulation; remains context-dependent

**Table 2:** Summary of AMP-based strategies explored for animal health applications.

AMP/Strategy	Species/Model	Main Outcome	Stage/Experimental Context
LLv	Broiler chickens	Enhanced immune indicators and jejunal immune gene expression	Dietary supplementation
lechonera	Broiler chickens challenged with necrotic enteritis	Mitigation of intestinal damage and microbiota imbalance	Feed supplementation/infection challenge
R7I	Geese infected with multidrug-resistant *Neisseria* S1	Reduced bacterial infection in vitro and in vivo	Oral AMP intervention/infection challenge
Epinecidin-1-expressing *Artemia*	Nile tilapia fry	Increased survival after bacterial challenge	Aquaculture functional feed
AMP mixture from chicken and pig intestines	Pengze crucian carp	Improved growth, immunity, antioxidant capacity and resistance to *Aeromonas hydrophila*	Aquaculture feed supplementation
PR39-expressing *Lactobacillus casei*	Mice challenged with enterotoxigenic *E. coli*	Improved intestinal morphology, immune status and survival	Engineered probiotic/infection challenge
KR32-expressing *Bacillus subtilis*	Piglets challenged with enterotoxigenic *E. coli*	Reduced oxidative intestinal injury and modulated faecal microbiota	Engineered probiotic/infection challenge
LfcinB-hLY	In vitro bacterial models/proposed livestock application	Recombinant production, antibacterial activity and digestive stability	Recombinant production/in vitro validation
MagII-CB	Bacterial models and *E. coli*-infected mice	Antibacterial activity and modulation of intestinal barrier and immune markers	Recombinant production/in vivo infection model

## Data Availability

No new data were created or analyzed in this study. Data sharing is not applicable to this article.

## Supplementary Materials

The following supporting information can be downloaded at: https://www.mdpi.com/article/10.3390/antibiotics15070649/s1, Table S1: Clinical trials focused on the antimicrobial peptide colistin. Data were acquired from ClinicalTrials.gov (https://clinicaltrials.gov/expert-search, accessed on 20 October 2025); Table S2: Clinical trials focused on the antimicrobial peptide C16G2. Data were acquired from ClinicalTrials.gov (https://clinicaltrials.gov/expert-search, accessed on 20 October 2025); Table S3: Clinical trials focused on the antimicrobial effect of specific antimicrobial peptides. Data were acquired from ClinicalTrials.gov (https://clinicaltrials.gov/expert-search, accessed on 20 October 2025); Table S4: Clinical trials using antimicrobial peptide levels as biomarkers. Data were acquired from ClinicalTrials.gov (https://clinicaltrials.gov/expert-search, accessed on 20 October 2025); Table S5: Clinical trials using LL-37 or cathelicidin antimicrobial peptide levels. Data were acquired from ClinicalTrials.gov (https://clinicaltrials.gov/expert-search, accessed on 20 October 2025).
