# Supplementary material for: Antimicrobial Peptides, Bacteriocins and Mycocins as Natural Antimicrobials: Applications in Food Safety, Agriculture and Healthcare
Source: Antibiotics (Basel). 2026 Jun 30;15(7):649. doi: 10.3390/antibiotics15070649 (PMC13403446; doi:10.3390/antibiotics15070649)
Supplement: Supplementary file 1 [file antibiotics-15-00649-s001.zip › antibiotics-4379832-supplementary.pdf]

Supplementary Materials

Antimicrobial Peptides, Bacteriocins and Mycocins as Natural Antimicrobials: Applications in Food Safety, Agriculture and Healthcare

Patrícia Branco [1,2,*], Elisabete Muchagato Maurício [1,*], Luís R. Raposo [1] and Catarina Roma-Rodrigues [1]

[1] BIORG—Bioengineering and Sustainability Research Group, Lusófona University, Av. Campo Grande 376, 1749-024 Lisbon, Portugal; luismrraposo@gmail.com (L.R.R.); catarina.rodrigues@ulusofona.pt (C.R.-R.)

[2] Linking Landscape, Environment, Agriculture and Food (LEAF), Associated Laboratory TERRA, Instituto Superior de Agronomia, University of Lisbon, Tapada da Ajuda, 1349-017 Lisbon, Portugal

* Correspondence: patricia.branco@ulusofona.pt (P.B.); elisabete.mauricio@ulusofona.pt (E.M.M.)

Table S1: Clinical trials focused on the antimicrobial peptide colistin. Data were acquired from ClinicalTrials.gov (https://clinicaltrials.gov/expert-search, accessed on 20 October 2025)

Colistin role in the study	Condition	Phase	Status (last update posted)	ClinicalTrials.gov ID / Reference (if applied)
Nephroprotective effect of curcumin in patients receiving colistin	Acute Kidney Injury<br>Drug-Induced Nephropathy	3	Recruiting<br>31-10-2023	NCT05613361
Effect of Colistin inhalation on ventilator associated pneumonia	Ventilator Associated Pneumonia	2<br>3	Completed<br>09-08-2018	NCT03622450
Effect of Aerosolised Colistin in Ventilator Associated Pneumonia	Ventilator Associated Pneumonia	4	Completed<br>17-02-2016	NCT02683603<br>[87]

Efficacy and Safety of Intravenous Sulbactam-ETX2514 in the Treatment of Patients that failed colistin or polymyxin B treatment	Acinetobacter Baumannii-calcoaceticus Complex<br>Hospital-acquired<br>Bacterial Pneumonia<br>Ventilator-associated Bacterial Pneumonia<br>Bacteremia<br>Colistin Resistant	3	Completed<br>01-02-2023	NCT03894046<br>[88]
Determination of the most empirical therapy of antibiotics, including Polymyxin AMP	Ventilation Acquired Pneumonia	3	Completed<br>18-12-2024	NCT06739382
Effect of plazomicin+colistin vs plazomicin+colistin+other antibiotic treatment	Bloodstream Infections (BSI) Due to CRE<br>Hospital-Acquired Bacterial Pneumonia (HABP) Due to CRE<br>Ventilator-Associated Bacterial Pneumonia (VABP) Due to CRE<br>Complicated Urinary Tract Infection (cUTI) Due to CRE<br>Acute Pyelonephritis (AP) Due to CRE	3	Completed<br>16-10-2018	NCT01970371<br>[89]
Effectiveness of an antibiotic combination that include Polymyxin B Sulphate	Eczema	3	Completed<br>24-02-2024	NCT01429701
Colistin monotherapy vs colistin and meropenem therapy	Gram negative bacterial infections	4	Completed<br>12-04-2017	NCT01732250<br>[90-93]
Effect of a cement loaded with colistin and erythromycin after total knee arthoplasty	Infection after primary total knee arthroplasty	n.a.	Completed<br>24-02-2016	NCT01631968<br>[94]
Effect of oral Colistin in eradication of carbapenem resistant *Klebsiella pneumonia*	Klebsiella Pneumoniae Carbapenemase Resistant Associated Bacteremia or Pneumonia	n.a.	Completed<br>28-04-2017	NCT01266499
Effect of Polymyxin B AMPs in adult patients with ventilator associated pneumonia due to carbapenem resistant *Acinetobacter baumannii*	Ventilator Associated Pneumonia	2	Completed<br>14-01-2025	NCT05685615
Effect of Colistin monotherapy vs colistin combined with a carbapenem in the treatment of drug-resistant Gram negative bacili	Pneumonia<br>Blood stream infection	3	Completed<br>14-11-2022	NCT01597973<br>[95]

Efficacy and Safety of Colistimethate Sodium (Colistin AMP) in The Treatment of Carbapenem-Resistant Enterobacteriaceae Infection	Carbapenem-Resistant Enterobacteriaceae Infection	n.a.	Recruiting 18-05-2025	NCT06051513 [96]
Effect of inhaled Colistin in prevention of ventilator associated pneumonia	Ventilator Associated Pneumonia	2 3	Not yet recruiting 10-06-2025	NCT06488794
Effect of Colistin AMP in pulmonary exacerbations	Cystic fibrosis	4	Completed 21-04-2023	NCT02918409
Clinical outcomes and safety of colistin AMP and tigecycline	Infection in ICU	4	Active, not recruiting 25-03-2025	NCT06893835
Effect of Colistin monotherapy vs Colistin combined treatment with tigecycline and vancomycin	Acinetobacter infections	n.a.	Completed 09-10-2015	NCT02573064 [97]
Pharmacokinetic and Pharmacodynamic Properties of Intravenous Colistimethate Sodium (Colistin)	colistin	n.a.	Completed 24-06-2019	NCT02288429
Colistin AMP monotherapy vs colistin-rifampin combination in drug-resistant *Acinetobacter Baumannii* infections	Acinetobacter infections	n.a.	Completed 09-08-2018	NCT03622918 [98]
Therapeutic strategies using combinations of colistin with other antibiotics	Bacterial infections Bacterial Sepsis	4	Recruiting 04-02-2025	NCT06440304
Colistin AMP vs Meropenem therapeutics in Ventilator-associated Pneumonia	Ventilator-associated bacterial pneumonia	3	Completed 03-02-2016	NCT01292031 [99,100]
Effect of colistin AMP in hospitalized COVID-19 patients with bacterial coinfection	Secondary bacterial infection in COVID-19 patients	3	Completed 19-01-2023	NCT05689229
Dosage of Colistin AMP in neonatal ICU patients with Gram-negative resistant infection	Colistin adverse reaction	n.a.	Recruiting 13-08-2025	NCT06472271
Effect of combination tobramycin and AMP colistin in reducing Pseudomonas growth in cystic fibrosis patients	Cystic fibrosis with pulmonary manifestations	3	Completed 14-11-2017	NCT03341741
Effect of colistin AMP in patients with haematological malignancies	Hematological infection	4	Completed 28-12-2017	NCT02966457 [101]

Pharmacokinetic study of colistin AMP in patients infected with multiresistant Gram-negative bacteria	Gram-negative bacteria infections	1	Completed 11-10-2016	NCT01060891 [102]
Colistin AMP monotherapy vs Colistin-fosfomycine therapy	Infection due to carbapenem resistant Acinetobacter	4	Recruiting 26-08-2024	NCT06570850
Pharmacokinetic study of intravenous colistin AMP in patients requiring low-efficiency dialysis	Bacterial infections	4	Completed 28-11-2022	NCT05586438
Effect of Colistin AMP dosage on clinical outcome of pediatric cancer patients	Gram-negative bacterial infections<br>Pediatric cancer<br>Colistin adverse reaction	4	Completed 16-09-2020	NCT03397914
Impact of Colistin in patients with carbapenem resistant Acinetobacter infections	Acinetobacter infections	n.a.	Completed 26-06-2018	NCT02482961 [103]
Comparative effect of Norfloxacin, Nitazoxanide and Colistin AMP as secondary prophylactic agents	Spontaneous bacterial peritonitis<br>Cirrhoses, liver<br>Ascites	4	Completed 14-02-2025	NCT06827756
Cefiderocol and Ampicillin-sulbactam vs. Colistin +/- Meropenem therapeutics	Carbapenem resistant bacterial infection<br>Acinetobacter bacteremia<br>Acinetobacter Pneumonia	4	Recruiting 01-08-2025	NCT05922124
Evaluation if Polymyxin AMP irrigation during pancreas surgery reduce organ space infections	Surgery<br>Infection	n.a.	Completed 23-02-2022	NCT02186457
Pharmacokinetic study of Polymyxin B in healthy and renal insufficient individuals	Antibiotic toxicity	1	Completed 27-04-2023	NCT05359627
Optimization of treatment regimen (including combinations with Colistin and Polymyxin B) for treatment of severe gram-negative bacterial infections	Bloodstream Infection<br>Ventilator Associated Bacterial Pneumonia<br>Hospital Acquired Bacterial Pneumonia<br>Carbapenem Resistant Bacterial Infection<br>Multidrug Resistance	4	Recruiting 04-06-2025	NCT07004049

Table S2: Clinical trials focused on the antimicrobial peptide C16G2. Data were acquired from ClinicalTrials.gov (https://clinicaltrials.gov/expert-search, accessed on 20 October 2025)

AMP role in the study	Condition	Phase	Status (last update posted)	ClinicalTrials.gov ID / Reference (if applied)
Microbiology, safety and tolerability of the AMP C16G2 varnish and strip	Dental caries	2	Completed 22-12-2022	NCT03196219
Microbiological activity of AMP C16G2	Dental caries	2	Completed 01-08-2029	NCT02594254
Safety and bacterial impact of AMP C16G2	Dental caries	n.a.	Completed 01-08-2019	NCT02509845
Safety and Microbiology of C16G2 varnish	Dental caries	2	Completed 29-12-2022	NCT03004365
Safety and microbiology of single or multiple oral C16G2 gel doses	Dental caries	2	Completed 01-08-2019	NCT02254993
Oral microbiology and safety of C16G2 AMP strip applications	Dental caries	2	Completed 22-12-2022	NCT03052842

Table S3: Clinical trials focused on the antimicrobial effect of specific antimicrobial peptides. Data were acquired from ClinicalTrials.gov (https://clinicaltrials.gov/expert-search, accessed on 20 October 2025)

AMP role in the study	Condition	Phase	Status (last update posted)	ClinicalTrials.gov ID / Reference (if applied)
Effect of AMP TAPS-18	Periodontitis	Early 1	Completed 18-11-2021	NCT05125718
Clinical efficacy of AMP PL-5 topical spray	Diabetic foot ulcers	2	Active, not recruiting 25-05-2025	NCT06189638
Safety, tolerability and PK profiles of AMP PL-18 in vaginal suppositories	Colpomycosis<br>Bacterial Vaginosis<br>Mixed Vaginitis	1	Recruiting 11-12-2023	NCT05340790
Effectiveness of a natural bioactive gel containing nisin (Sterify gel®)	Peri-implantitis<br>Dental implants<br>Non-surgical periodontal therapy	n.a.	Recruiting 22-08-2025	NCT07088679

Table S4: Clinical trials using antimicrobial peptide levels as biomarkers. Data were acquired from ClinicalTrials.gov (https://clinicaltrials.gov/expert-search, accessed on 20 October 2025)

AMP role in the study	Condition	Phase	Status<br>(last update posted)	ClinicalTrials.gov ID /<br>Reference<br>(if applied)
Levels of faecal AMPs (calprotectin and LL-37) as outcome measure of ingestion of a probiotic	Safety and efficacy of a probiotic	n.a.	Completed<br>29-08-2025	NCT06779994
Levels of AMP hCAP18 after ingestion of Vitamin D and Omega-3 fatty acids	Infections<br>Levels of hCAP18	n.a.	Active, not recruiting<br>25-09-2025	NCT01758081
Increased secretion of AMPs in gastrointestinal tract after aminoacid fortified oral rehydration therapy	Acute Gastroenteritis	1<br>2	Recruiting<br>10-06-2024	NCT06137014
AMP levels in the skin of patients with Rosacea	Rosacea	Early 1	Completed<br>13-09-2019	NCT01398280
AMP levels in skin explants after pimecrolimus administration	Atopic dermatitis	n.a.	Completed<br>29-03-2017	NCT00379678
Vaginal AMP profile	Stress urinary incontinence<br>Prolapse	n.a.	Completed<br>22-01-2019	NCT02605135
Levels of AMP in skin of patients prior, during and after treatment with Secukinumab	Psoriasis vulgaris	n.a.	Completed<br>16-08-2019	NCT03149900
Role of AMPs in host defence against Vaccinia Virus	Atopic dermatitis	n.a.	Completed<br>18-10-2016	NCT00407069
AMP levels after treatment with oestrogen	Overactive bladder<br>Incontinence<br>Nocturia	4	Completed<br>23-09-2021	NCT02835846<br>[108]
Impact of oestrogen in AMP levels	Overactive bladder	n.a.	Completed<br>15-04-2021	NCT02524769<br>[108]
Identification of AMPs in periodontitis	Periodontal disease	n.a.	Completed<br>30-03-2017	NCT02793453

	Chronic periodontitis			
Expression of AMPs at the level of the periodontium	Cystic fibrosis<br>Periodontitis<br>Gingivitis	n.a.	Recruiting<br>15-11-2023	NCT06133335
Effect on the immune response of therapeutic strategy based on photodynamic therapy alone or in combination of AMPs	Hidradenitis Suppurativa	n.a.	Not yet recruiting<br>31-10-2023	NCT05208099
Evaluation of AMPs in Psoriasis etiopathogenesis	Psoriasis	n.a.	Completed<br>23-03-2018	NCT03475914
Evaluation of effect of AMPs application after non-surgical periodontal therapy	Periodontitis	4	Completed<br>30-04-2024	NCT05530252
Effect of endogenous AMPs in acute graft-versus-host disease	Acute GVHD<br>Ulcerative colitis<br>Morbus Chron	n.a.	Completed<br>18-12-2024	NCT04522843
Levels of AMPs in faeces after ingestion of *Bifidobacterium longum* subsp. *infantis* BI45 as a food supplement	Healthy	n.a.	Not yet recruiting	NCT06863415
Levels of AMPs in skin after contact with petrolatum	Skin disease	n.a.	Completed<br>16-05-2017	NCT02338076
Expression of AMPs in rosacea after treatment with topical Ivermectin 1% cream	Rosaceae	1<br>2	Completed<br>05-10-2021	NCT02806414
Difference between the AMPs in inner-colonic samples in comparison of spontaneously excreted stool samples	Irritable Bowel disease	n.a.	Completed<br>27-04-2023	NCT05640583
Levels of AMPs following bacteraemia after non-surgical periodontal treatment	Bacteremia<br>Periodontal diseases<br>Sepsis<br>Heart diseases	n.a.	Active, not recruiting	NCT04565691
Fecal Intestinal AMPs Secretion after oral HA35 supplementation	Gastrointestinal microbiome	n.a.	Completed<br>03-09-2020	NCT02867605
Levels of nasal AMPs after vitamin D supplementation in patients with COPD	COPD	n.a.	Completed<br>10-09-2019	NCT02122627<br>[109,110]

Table S5: Clinical trials using LL-37 or cathelicidin antimicrobial peptide levels. Data were acquired from ClinicalTrials.gov (https://clinicaltrials.gov/expert-search, accessed on 20 October 2025)

AMP role in the study	Condition	Phase	Status (last update posted)	Clinicaltrials.gov ID / Reference (if applied)
Skin AMP expression alteration as outcome measure of vitamin D3 ingestion.	Atopic dermatitis / Psoriasis	2	12-04-2017	NCT00789880
Levels of AMP LL-37 after ingestion of vitamin D and phenylbutyrate	Pulmonary tuberculosis	2	Completed 13-02-2015	NCT01580007 [111-113]
Correlation of Levels of AMP LL-37 and levels of vitamin D in peri-implant sulcus fluid	Peri-implantitis and peri-implant mucositis		Completed 10-03-2025	NCT06867250
Dosage and effect on immune system of the AMP LL37	Melanoma	1<br>2	Completed 09-12-2021	NCT02225366
AMP expression during high-dose vitamin D supplementation	Respiratory failure	2	Completed 09-01-2017	NCT01372995 [114]
Levels of AMP LL37 after high dose Vitamin D	Sepsis	4	Completed 07-11-2022	NCT05244018
AMP LL-37 levels in passive smoking children	Innate immunity<br>Periodontal health	n.a.	Completed 21-08-2018	NCT03639376
Levels of AMP LL-37 to evaluate the effect of probiotics *Limosilactobacillus fermentum* LF61	Intestinal health	n.a.	Not yet recruiting 12-03-2025	NCT06873425
Role of anti-inflammatory cytokines and AMP LL-37	Periodontal disease<br>Periodontitis	n.a.	Completed 30-09-2022	NCT04404335 [115]
Levels of AMPs LL-37 after treatment with soaps	healthy	n.a.	Completed 02-10-2019	NCT01951352
Levels of AMP cathelicin and LL-37 after inhalation of corticosteroid and/or diesel exhaust exposure	Chronic obstructive pulmonary disease	4	Not yet recruiting 27-03-2025	NCT06552364
Levels of AMPs in saliva after maternal pre- and postnatal vitamin D therapy	Vitamin D deficiency	n.a.	Completed 06-09-2017	NCT03273725

	Dental carries<br>Dental enamel hypoplasia			
Salivary levels of AMP LL-37 and oxidative stress markers	Passive smoking<br>Oxidative stress<br>Dental carries<br>Children	n.a.	Completed<br>03-03-2020	NCT04292548
Influence of keto diet and intermittent fasting on the levels of AMP LL-37	Gingivitis	Early 1	Completed<br>19-09-2024	NCT06597708
Level of cathelicidin AMP after supplementation with cholecalciferol in dialysis patients	Chronic kidney disease	2	Completed<br>14-03-2025	NCT01974245<br>[116]
Correlation between levels of Cathelicidin and vitamin D in patients with COPD	COPD<br>COPD moderate<br>COPD severe	4	Completed<br>12-04-2024	NCT05431218

AMP – antimicrobial peptide; COPD – chronic obstructive pulmonary disease; CRE – Carbapenem-resistant *Enterobacteriaceae*; LEAP 2 - liver enriched antimicrobial peptide 2; MRSA – methicillin resistant *Staphylococcus aureus*; n.a. – not applicable
